# Shifting Shapes: The Endothelial-to-Mesenchymal Transition as a Driver for Cancer Progression

**DOI:** 10.3390/ijms26136353

**Published:** 2025-07-01

**Authors:** Lucia Giordanengo, Alessia Proment, Virginia Botta, Francesca Picca, H. M. Waqas Munir, Jiahao Tao, Martina Olivero, Riccardo Taulli, Francesca Bersani, Dario Sangiolo, Silvia Novello, Giorgio Vittorio Scagliotti, Alessandra Merlini, Gabriella Doronzo

**Affiliations:** 1 Department of Oncology, Translational Oncology Laboratory “Paola Gilardi”, San Luigi Gonzaga University Hospital, University of Turin, 10043 Orbassano, Italy; lucia.giordanengo@unito.it (L.G.); alessia.proment@unito.it (A.P.); virginia.botta@unito.it (V.B.); francesca.picca@unito.it (F.P.); hafizmuhammadwaqas.munir@unito.it (H.M.W.M.); jiahao.tao@unito.it (J.T.); martina.olivero@unito.it (M.O.); riccardo.taulli@unito.it (R.T.); francesca.bersani@unito.it (F.B.); dario.sangiolo@unito.it (D.S.); silvia.novello@unito.it (S.N.); giorgio.scagliotti@unito.it (G.V.S.); alessandra.merlini@unito.it (A.M.); 2 Candiolo Cancer Institute-IRCCS-FPO, 10060 Candiolo, Italy; 3 Molecular Biotechnology Center “Guido Tarone”, University of Turin, 10126 Turin, Italy; 4 Division of Medical Oncology, San Luigi Gonzaga University Hospital, 10043 Orbassano, Italy

**Keywords:** endothelial cells, endothelial-to-mesenchymal transition (EndMT), tumor microenvironment, immune escape, metastasis, resistance to therapy

## Abstract

Endothelial-to-mesenchymal transition (EndMT) is a dynamic cellular process characterized by a phenotypic-functional switch of cells from endothelial-to-mesenchymal traits. Many studies have identified EndMT as a key driver of tumor growth and progression. EndMT supports tumor cell proliferation by creating a tumor microenvironment that facilitates cancer cell survival. Notably, EndMT is an important source of cancer-associated fibroblasts, leads to immune dysregulation and immune escape, and supports metastasis and resistance to therapy. Hence, understanding the intricate relationship between EndMT and cancer progression offers exciting new avenues for therapeutic intervention. This review aims to describe the central role of EndMT in tumor progression, highlighting the molecular mechanisms underlying this endothelial alteration and its significant involvement at all stages of tumor progression.

## 1. Introduction

The tumor microenvironment is a complex *milieu* of heterogeneous stromal cells, including vascular cells (e.g., endothelial cells, smooth muscle cells, pericytes), able to interact with each other to support tumor initiation, growth, and progression [1]. Different studies underline how tumor endothelial cells (ECs) are a very dynamic cell type characterized by high phenotypic and functional plasticity, defined as the functional and phenotypic flexibility of ECs, enabling them to adapt, transition, and contribute to various physiological and pathological processes. This process is in part related to an intense, specific crosstalk with parenchymal tumor cells and other tumor-associated stromal cells [2,3].

The most extreme form of endothelial plasticity is endothelial-to-mesenchymal transition (EndMT), a transdifferentiation process during which ECs lose their specific endothelial key features and assume a mesenchymal phenotype undergoing profound morphological, functional, genetic, and molecular changes [4,5,6].

**Figure 1:**
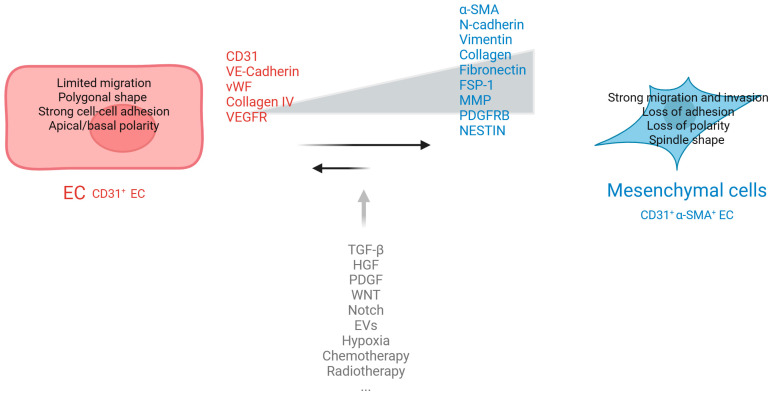
**EndMT markers and specific cellular alteration.** During the EndMT, cells show a reduced expression of typical endothelial markers such as VE-cadherin, CD31, TIE1, TIE2, and vWF. At the same time, there is an increased expression of mesenchymal markers, including FSP-1, αSMA, N-cadherin, vimentin, fibronectin, type I, and type III collagen, as well as the enzymes MMP-2 and MMP-9. This process also involves alterations in cell shape and polarity, a breakdown of cell–cell connections, and enhanced cell mobility. Transforming growth factor-beta (TGF-β), hepatocyte growth factor (HGF), neurogenic locus notch homolog protein (Notch), platelet-derived growth factor (PDGF), extracellular vesicles (EVs), Von Willebrand factor (vWF), vascular endothelial growth factor receptor (VEGFR), tyrosine kinase receptor-2 (Tie-2), platelet endothelial cell adhesion molecule 1 (PECAM1), α-smooth muscle actin (α-SMA), fibroblast-specific protein-1 (FSP-1), matrix metalloproteinase (MMP), fibroblast activation protein (FAP). Created with BioRender.com.

The acquisition of a mesenchymal phenotype is characterized by the loss of cellular adhesions and cytoskeleton reorganization, which sustains the alteration of cell polarity enhancing cell migration and invasiveness [4,5,6]. ECs lose endothelial specific markers, such as von Willebrand factor (vWF), platelet endothelial cell adhesion molecule-1 (PECAM-1; also known as CD31), vascular-endothelial cadherin (VE-cadherin), vascular endothelial growth factor receptor 2 (VEGFR2), Tie1-2, and ZO-1, and start synthetizing mesenchymal protein markers, including alpha-smooth muscle actin (α-SMA), fibroblast activation protein (FAP), vimentin, fibronectin, N-cadherin, fibroblast specific protein 1 (FSP1), and collagen type I [4,5,6,7] (Figure 1).

EndMT is modulated by several molecules and pathways that vary based on the tissue type, the stroma composition, and tumor progression. EndMT [8] is a progressive process with intermediate phenotypes: it can be transient and reversible if the stimulus decreases shortly after its initiation, or it becomes stable and irreversible if chronic induction is present [8,9]. Early descriptions of EndMT suggested it to be a one-way transformation. However, recent studies have revealed that EndMT is dynamic and reversible, existing along a continuum of phenotypic states rather than operating as an all-or-nothing process. Many ECs undergoing EndMT adopt intermediate or partial phenotypes, retaining features of both lineages. These hybrid states are particularly prevalent during angiogenesis, providing strong evidence for the existence of intermediary phases within the EndMT spectrum [10]. This partial transition has been observed in developmental and pathological contexts, including tumor growth, supporting the model of EndMT as a spectrum of intermediate states rather than a terminal endpoint. Importantly, these intermediate EndMT states are metastable; when EndMT-inducing signals (such as TGF-β or hypoxia) are removed, the cells can revert to an endothelial phenotype.

Increasing studies identify EndMT-related processes as an important trigger for several alterations characterizing the tumor microenvironment such as the development of cancer-associated fibroblasts (CAFs), aberrant tumor vessels, and fibrosis [11,12,13]. In the tumor context, the EndMT process was first identified in melanoma [14] and then in Kaposi’s sarcoma [15], pancreatic cancer [16], hepatocellular carcinoma [17], esophageal adenocarcinoma [18], breast cancer [19], glioblastoma [12], lung cancer [20], and colon cancer [21]. EndMt was not described in the corresponding healthy tissue [15,17,22]. Very interestingly, EndMT is also described for circulating tumor endothelial cells: double-positive circulating tumor endothelial cells (CD31+-vimentin+) were found in blood samples from patients with non- small-cell-lung cancer (NSCLC) [23].

EndMT leads to cancer progression, influencing tumor heterogeneity, growth, metastasis, immune-escape, and resistance to treatment [4,5,6,7,8]. Finally, EndMT has remained relatively underappreciated so far in most studies focusing on tumor progression, notwithstanding emerging evidence on its key role in the process. For all these reasons, recognizing EndMT as a key player in tumor progression marks a crucial step toward a more comprehensive understanding of cancer dynamics and the development of more effective treatments.

## 2. EMT and EndMT Interplay in Cancer Progression

Epithelial–mesenchymal transition (EMT) [24,25,26,27] and EndMT [4,5,6,7,8,9,10,11] are fundamental physiological processes involved in embryonal development, cardiac morphogenesis, and wound healing. Both are also involved in pathological settings such as cancer progression, during which they contribute to fibrosis, tumor microenvironment shaping, metastasization, immune evasion, and prompt resistance to treatments [4,5,6,7,8,9,10,11,24,25,26,27]. The mesenchymal transition leads to the disruption of cell polarity, enhanced cellular mobility, and acquisition of mesenchymal traits, contributing to the invasive behavior of cancer cells and associated stromal components like tumor ECs [4,5,6,7,8,9,10,11,24,25,26,27]. EMT involves the transformation of epithelial cells into a mesenchymal phenotype, leading to increased motility, invasiveness, and resistance to apoptosis. This process enables tumor cells to detach from the primary site, invade surrounding tissues, and eventually metastasize to distant organs. In parallel, EndMT is characterized by the loss of endothelial markers and the acquisition of mesenchymal traits by ECs. This transition contributes to the remodeling of the tumor microenvironment by promoting fibrosis, abnormal angiogenesis, and the generation of CAFs, which in turn support tumor growth and invasion. While EMT facilitates the dissemination of tumor cells, EndMT alters the tumor stroma and vasculature, creating a permissive environment for cancer cell migration, immune evasion, and metastatic colonization. The molecular mechanisms of EMT and EndMT are also highly similar. EMT and EndMT are both triggered by a similar group of pro-inflammatory and pro-fibrotic factors, with TGF-β being the most prominent, as it plays a key role in initiating and maintaining the mesenchymal transition [4,5,6,7,8,9,10,11,24,25,26,27]. On the intracellular level, both transitions rely on overlapping signaling cascades [4,5,6,7,8,9,10,11,24,25,26,27], such TGF-β/Smad, Notch, Wnt/β-catenin, and PI3K/AKT. These signals activate a core group of transcription factors, SNAIL, SLUG, TWIST, and ZEB1/2, that suppress the expression of adhesion molecules like E-cadherin in epithelial cells and VE-cadherin in Ecs. Finally, EMT and EndMT are characterized for the gain of mesenchymal markers such as FSP-1, α-SMA, fibroblast activation protein (FAP), collagen type I-III, and N-cadherin [4,5,6,7,8,9,10,11,24,25,26,27]. On the other hand, other cellular-type specific markers are a loss such as CD31 also known as PECAM1, vWF, and Tie-2 [4,5,6,7,8,9,10,11,24,25,26,27].

Taking all these factors into account, EMT and EndMT are deeply interconnected processes that occur in parallel within the tumor microenvironment to sustain tumor progression. This close interplay between “bad transitions” also complicates the development of targeted therapies, as interfering with one process may inadvertently affect the other. This intricate crosstalk underscores the complexity of mesenchymal transitions in cancer and highlights the need for continued scientific research to understand their mechanisms and develop effective, selective therapeutic strategies.

The table below (Table 1) highlights the key similarities and differences between EMT and EndMT. For a more comprehensive overview of EMT-specific characteristics, we recommend consulting recent, high-quality reviews on the topic [24,25,26,27].

## 3. KEY Actors in EndMT

**Figure 2:**
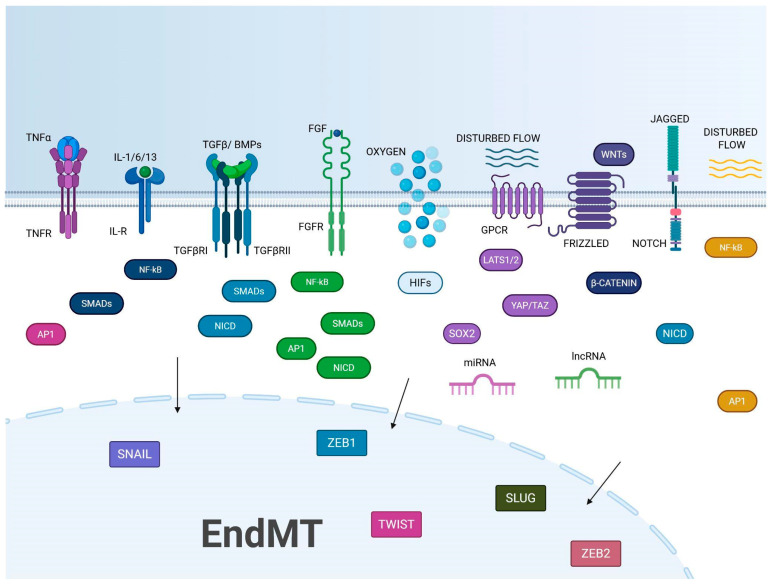
**Schematic representation of key regulators and intracellular signaling pathways involved in EndMT.** The figure illustrates the several major molecular regulators of EndMT, including extracellular signals such as TGF-β, inflammatory cytokines, hypoxia, and stress which activate intracellular pathways (e.g., SMAD, PI3K/Akt, Notch, MAPK). Downstream transcription factors such as Snail, Slug, Twist, and ZEB1/2 are shown promoting the EndMT process by repressing endothelial markers and inducing mesenchymal gene expression. The roles of regulatory non-coding RNAs, including specific miRNAs and lncRNAs, are also highlighted as modulators of gene expression during EndMT. Created with BioRender.com.

In vitro and in vivo studies performed on ECs from different tissues, including both human and mouse, demonstrate that EndMT is supported by the physical proximity of ECs with tumor and stromal cells as well as by soluble factors released by the same cells. Among these, key players in EndMT include TGF-β [28,29,30,31,32], Notch [19,33], Wnt/β-catenin [34,35], alpha-1-antitrypsin (A1AT) [36], HGF/c-Met signaling [12], platelet-derived growth factor (PDGF) pathway [37], inflammatory cytokines such as Interleukin- (IL)-1 and -6 [18,38,39], and tumor necrosis factor-alpha (TNF-α) [40,41]. Several studies also highlight the role of oxidative stress [42] and hypoxia [43,44] as triggers of EndMT. Finally, therapeutic interventions involving, in particular, radiotherapy [20,45,46,47], and systemic factors can be included among the putative inducers of EndMT during tumor progression. The multifactorial regulation of EndMT, driven by multiple signaling cascades, epigenetic modifications, and microenvironmental influences, highlights the need for further in-depth research to fully understand its role in cancer progression (Figure 2).

*TGF-β.* Numerous studies have shown that TGF-β growth factors are essential for the initiation and progression of EndMT [18,28,29,30,31,32,48,49,50,51]. This role was evidenced by observing that the number of mesenchymal cells derived from ECs is reduced after impairing the TGF-β signaling in transgenic mice. In particular, EndMT is inhibited by using a TGF-β receptor kinase inhibitor or by employing small molecule inhibitors that target the TGF-β-mediated intracellular phosphorylation pathway [52,53,54,55]. The TGF-β cytokine family includes two main subfamilies: bone morphogenetic proteins (BMPs) and the TGF-β/activin A subfamilies. TGF-β is secreted as an inactive form bound to latency-associated peptide (LAP) and is activated by the interplay with αvβ1 and αvβ6 integrins [51]. In ECs, TGF-β interacts as a homodimer with TGF-β receptors I and II, leading to autophosphorylation of TGF-βII receptor, phosphorylation of TGF-βRI, and formation of an active receptor complex capable of phosphorylating specific serine residues on Smad2 and Smad3 proteins. These phosphorylated Smads form a complex with Smad4 and translocate to the nucleus, where they activate the Smad-binding elements (SBE) in the promoter regions of TGF-β-responsive genes, triggering their transcription. In contrast, Smad6 and Smad7 act as negative regulators inhibiting TGF-β signaling [51]. A non-canonical pathway involves mitogen-activated protein kinase (MAPK) family, PhosphatidylInositol 3-Kinase (PI3K), RHO and RAC GTPase, Abelson tyrosine kinase (c-ABL), and Protein Kinase C- δ (PKC-δ). Through all these pathways, TGF-β stimulates the activity of SNAIL, SLUG, and Twist-related protein-1 (TWIST) transcription factors thereby up- and downregulating the expression of mesenchymal and endothelial markers respectively [51]. Several molecules are capable of modulating TGF-β1-induced EndMT: Sirtuin-1 and 3 (SIRT1 and SIRT3) proteins act as inhibitors [56,57]; Endothelin-1 (ET-1), a potent vasoconstrictor, increases the expression of SNAIL and TWIST; TGF-β1 and its receptors create an autocrine loop that amplifies EndMT [58,59]; Caveolin-1 (CAV-1) plays an essential role in the internalization, trafficking, and degradation of TGF-β receptors, thereby influencing intracellular signaling [60]. Studies show that the downregulation of Signal Activator of Transcription 3 (STAT3) in response to inflammation contributes to fibrotic processes in EndMT [61]. Moreover, as detailed further below, micro ribonucleotide acids (miRNAs) and long non-coding RNAs (lncRNA) are also involved in the control of TGF-β-mediated EndMT.

The TGF-β cytokines are often overexpressed in tumors: TGF-β1 induces EndMT in melanoma [62] and hepatocellular carcinoma [63], and TGF-β2 in esophageal carcinoma [18] and invasive colon carcinoma [21]. Via the PLEK2–SHIP2 pathway, TGF-β promotes both lung cancer cell migration and vascular invasion in NSCLC [64].

*Notch.* Studies in lung cancer [65], glioblastoma (GBM) [66], and breast cancer [19] suggest an essential crosstalk between Notch and TGF-β1 to support EndMT.

The Notch receptor consists of an extracellular and an intracellular domain (NICD) which, after the interaction with ligands, is cleaved by γ-secretase, and translocates to the nucleus. There, NICD binds to the CSL transcriptional repressor complex, leading to the activation of genes involved in tumor progression, such as NF-κB, AKT, and p21. Aberrant activation of Notch signaling can promote EndMT particularly in the context of vascular remodeling and fibrosis. Notch cooperates with TGF-β to drive the expression and stabilization of SNAIL, both directly and indirectly, by inducing hypoxia-inducible factor 1α (HIF-1α), which promotes the transcription of lysyl oxidase (LOX) [67]. In addition, inhibition of the Jagged-1 (Jag1)-Notch pathway leads to an increase in CXC chemokine receptor type 7 (CXCR7) levels in TGF-β1-treated ECs via the Smad2/Smad3 pathway. For this reason, CXCR7 may act as a feedback mechanism to control Notch signaling and regulate TGF-β-induced EndMT and fibrosis [68]. Inhibition of canonical Notch signaling accelerates EndMT during certain stages of wound healing and scar formation, resulting in excessive tissue fibrosis and increased TGF-β1 expression [69].

*Wnt/β-catenin pathway.* It is known that the Wnt/β-catenin pathway supports EndMT in oral squamous carcinoma [70] and in GBM [71]. Wnt signaling modulates β-catenin, a protein essential for cell adhesion, cell proliferation, survival, stemness, invasion, and resistance to apoptosis. Wnt proteins exert their effects by binding Frizzled receptors and LRP5/6 co-receptors. Normally, β-catenin is degraded by the proteasome via the APC, Axin, and GSK-3β complex. In contrast, after the activation of Wnt signaling and destruction of APC/Axin/GSK-3β complex, β-catenin accumulates in the cytoplasm and translocates into the nucleus where it binds the TCF/LEF transcription factor family, enhancing cell survival, proliferation, adhesion, and EndMT [72]. The inhibitory role of GSK-3β in EndMT was demonstrated using a tumor spheroid model containing lung cancer cells and HUVECs. Furthermore, a synergistic anti-cancer effect was observed when GSK-3β modulation was combined with gefitinib, an EGFR inhibitor [65].

*Oxidative stress and ROS.* Oxidative stress and reactive oxygen species (ROS) are recognized as key factors in inducing EndMT [42]. ECs exposed to increasing concentrations of hydrogen peroxide (H2O2) undergo a dose-dependent transition to mesenchymal cells driven by increased endogenous TGF-β production and activation of Smad3, p38, and NF-κB pathways [42].

The nicotinamide adenine dinucleotide phosphate (NADPH) oxidase system activates EndMT. In particular, TGF-β induces (NOX)-4 and hyperglycemia enhances NOX4-mediated ROS production, which activates the TGF-β1-Smad2/3 pathway, leading to EndMT and ECs fibrosis/apoptosis [73]. Antioxidants that scavenge reactive ROS, such as N-acetylcysteine, or inhibitors of ROS production, such as apocynin, or physical interventions, such as intensity-pulsed ultrasound (LIPUS), can prevent endotoxin-induced EndMT [74,75].

*Hypoxia.* Several studies suggest that hypoxia is a potent inducer of EndMT, with HIF-1α playing a key role in mediating this effect [44,76,77,78]. Hypoxia acts alongside different pathways involved in EndMT [44,76,77,78]. In ECs, hypoxia leads to an increased release of TGF-β1, activating the ALK5/SMAD2 pathway. This signaling cascade results in the downregulation of EC markers (such as VE-cadherin) and the upregulation of mesenchymal markers (such as α-SMA and FSP1). Furthermore, hypoxia amplifies canonical TGF-β signaling, boosting transcription factors such as SNAIL, Slug, and Zeb1, which reinforce mesenchymal programming in ECs [76,77,78]. The hypoxic environment also activates interconnected pathways including Notch, Wnt/β-catenin, and NF-κB, which synergize with HIF signaling to drive EndoMT. In addition, RHOJ depletion reduces hypoxia-induced EndMT by preventing the binding of the transcriptional repressors TWIST and SNAIL to EC-specific gene promoters. Moreover, HIF-2α induces increased expression of SNAIL-1/2, promoting a strong mesenchymal transition [44,76,77,78].

*Cytokines.* Inflammation is a key driver of EndMT, able to activate signaling pathways that downregulate endothelial markers and promote mesenchymal traits. In the tumor microenvironment, this inflammation-induced EndMT contributes to abnormal vasculature, immune evasion, and the generation of cancer-associated fibroblasts, ultimately supporting tumor progression and therapeutic resistance. Synergistically with TGF-β, inflammatory cytokines like IL-1β, IL-6, IL-13, IFN, and TNF-α are play a crucial role in the regulation of immune response, inflammation, and tissue repair, and they contribute to the induction of EndMT [79,80]. They are produced by macrophages, monocytes, dendritic cells, neutrophils, mast cells, and eosinophils, but also by ECs, fibroblasts, epithelial cells, and smooth muscle cells. NF-κB seems to be a critical factor in cytokine-induced EndMT. Co-stimulation with IL-1β and TGF-β2, or stimulation with TNF-α and IL-6 can trigger EndMT via NF-κB activation. These results highlight NF-κB as a central mediator of EndMT under inflammatory conditions [79,80].

Treatment of human dermal microvascular ECs with IL-1β lead to significant phenotypic changes associated with EndMT as well as the loss of endothelial-specific functions, such as the ability to form tubular structures essential for angiogenesis [81].

The involvement of IL-6 and TNF-α via the Akt/NF-κB and canonical TGF-β pathways is first observed in three-dimensional in vitro cultures of both embryonic and adult ECs. The IL-6 effect is mediated by the activation of its receptor by L1CAM, a transmembrane glycoprotein, and by the IL-6R-mediated STAT phosphorylation [38].

Also, IL-13 strongly induced EndMT indirectly via the downregulation of miR-424/503 and the increased expression of Rictor, a key component of the mTOR pathway [82].

IFN increases the synthesis of TGF-β2 and SNAI1 and supports the downregulation of VE-cadherin and the alteration of the actin cytoskeleton [83].

Among the chemokines, macrophages can induce the expression of C-C motif chemokine ligand 4 (CCL4), thereby enhancing endothelial permeability and monocyte adhesion. CCL-4 is specifically expressed by M1-type macrophage-derived foam cells (M1-FCs), but not by M2-FCs. CCL-4 exerts its effects by activating its receptor, CCR-5, which in turn upregulates TGF-β. Inhibiting CCR-5 signaling or silencing TGF-β could reverse CCL-4-induced EndMT [84].

EndMT is triggered by the co-culture of ECs with esophageal adenocarcinoma cells expressing high levels of IL-1β and TGF-β2 [85].

*Growth factors.* Several growth factors are known to negatively regulate TGF-β signaling, thereby inhibiting the EndMT. In particular, a key role was attributed to fibroblast growth factor-2 (FGF2) and vascular endothelial growth factor (VEGF).

FGF2 is involved in a wide range of biological processes, including cell growth, wound healing, angiogenesis, and tissue development. It inhibits TGF-β-induced EndMT in various ECs, including aortic, dermal, lymphatic, and umbilical vein ECs [86,87,88]. The mechanisms through which FGF2 inhibits EndMT involve multiple signaling pathways, including Smad2 in a Ras-MAPK-dependent manner, ALK5, TGFBR2, and SARA via miR-20a [88]. High-throughput studies have also identified FGF2 as a potent inhibitor of TGF-β-induced EndMT in ovine mitral valve Ecs [89]. Furthermore, FGF2 influences EndMT by regulating microRNAs, particularly let-7, which inhibits TGF-β signaling [86]. Inflammatory conditions involving IFN-γ and TNF-α impair FGF2 actions by downregulating let-7 expression or FGFR1 [86]. Furthermore, the activation of the MEK/ERK pathway by FGF2 contributes to its anti-EndMT effect by promoting ECs proliferation and survival, and counteracting TGF-β-induced EndMT [90].

VEGF is a key regulator of angiogenesis and it is involved in the formation of new blood vessels. It promotes EC proliferation, migration, and survival, and plays an essential role in both physiological processes, such as wound healing, and pathological processes, such as cancer and diabetic retinopathy, which involve vascular growth. By counteracting the pro-EndMT role of Notch1, VEGF suppresses TGF-β-induced EndMT in ovine aortic valve ECs [91]. Furthermore, VEGF exerts protective effects against EndMT-driven fibrosis in the kidney and heart [92,93].

Hepatocyte growth factor (HGF) is a multifunctional growth factor that can promote cell proliferation, motility, survival, and morphogenesis. It exerts its effects by binding to its receptor, c-Met, which is found in various cell types, including epithelial and ECs. HGF plays a particularly important role in tissue regeneration, angiogenesis, and wound healing. It also exhibits anti-inflammatory and anti-fibrotic properties, counteracting the effects of TGF-β and inhibiting EMT and EndMT. Treatment with HGF inhibits the TGF-β-induced Smad and Akt/mTOR signaling pathways in HUVECs and human renal glomerular ECs, reducing the progression of EndMT triggered by TGF-β1, including cell transdifferentiation and migration [94].

BMP-7, a member of the TGF-β superfamily, is primarily responsible for bone and cartilage development, organogenesis, and tissue repair. It plays a critical role in embryonic development and its regenerative effects in the kidney, eye, and other organs have been studied. Several studies have demonstrated how BMP-7 regulates and inhibits EndoMT in various pathological fibrosis contexts. In vitro studies of human pulmonary microvascular ECs suggested that there is inhibitory crosstalk between BMP-7 and TGF-β1. TGF-β1 reduces BMPR2 expression, but treatment with BMPs restores the balance between BMPR2 and TGF-β and blocks TGF-β-induced EndMT [52]. Via in vitro and in vivo studies based on hypoxia-induced pulmonary arterial hypertension (PAH) and pulmonary artery ECs, the role of BMP-7 as inhibitor of hypoxia-induced EndMT and cell migration was demonstrated. BMP-7 acts via the modulation of the mTOR pathway [95].

*Epigenetic regulation, the role of miRNAs and lncRNAs.* Different studies underline the role of the epigenetic regulation of EndMT and, in particular, of MicroRNAs (miRs) and long non-coding RNAs (lncRNAs). miRs are short RNA sequences, typically around 22 nucleotides long, that do not code for proteins but help control gene expression by attaching to mRNAs and either blocking their translation or causing their breakdown [96,97,98]. LncRNAs are RNA molecules longer than 200 nucleotides that, despite not producing proteins, influence gene activity through various mechanisms, such as modifying chromatin structure, regulating transcription and RNA splicing, or binding to miRNAs to affect their function. By targeting and modulating the transcription of essential genes involved in EndMT, miRNAs and lncRNAs influence the downregulation of endothelial markers and the upregulation of mesenchymal markers. Their regulatory function affects all major cellular changes of EndMT such as loss of cell–cell junctions, altered polarity, and increased cell motility, highlighting miRNAs.

The TGF-β signaling pathway is a major inducer of EndMT and has been shown to downregulate key miRNAs such as miR-200a, miR-20a, miR-29, and miR-630, thereby promoting EndMT [96,97,98]. The miR-200 family, particularly miR-200a, plays a critical inhibitory role by targeting GRB2 and suppressing mesenchymal markers like FSP-1 and α-SMA while promoting endothelial markers such as VE-cadherin and PECAM-1 [96,99,100]. Similarly, miR-20a is downregulated by TGF-β1, and its overexpression inhibits EndMT by targeting TGF-βR1-2, and SARA [99]. Other miRs, like miR-630, directly target transcription factors such as Slug, suppressing EndMT induced by TGF-β and BMP-4 in endothelial cells [97]. MiR-29 is also reduced under TGF-β influence and is linked to the regulation of both endothelial and mesenchymal markers, potentially affecting DPP-4 expression in diabetic kidney tissue [98].

As previously described, FGF modulates TGF-β signaling across different cell types [101,102]. Importantly, endothelial FGF signaling appears to counteract TGF-β-induced EndMT by also modulating the expression of specific miRNAs, such as let-7 and miR-20a [86,88]. For example, knockdown of FRS2, a key FGF signaling adaptor, reduces let-7 levels and increases TGFβR1 expression, promoting EndMT and neointima formation in human umbilical artery ECs [86]. Similarly, treatment with TGF-β2, IL-1β, and TNF-α leads to decreased FGFR expression and reduced let-7 levels, contributing to EndMT [86]. In addition, FGF-2 upregulates miR-20a, which in turn suppresses TGFβR1, TGFβR1, and SARA expression, thereby inhibiting EndMT [88]. miR-148b inhibition promotes EndMT in vitro in HUVECs [103] via the downregulation of TGFβ-2 and SMAD2 gene expression.

Other miRNAs have been identified as important regulators of TGF-β-induced EndMT. miR-23 inhibits EndMT in mouse embryonic ECs by directly targeting Has2, a gene involved in cardiac valve development [104]. miR-532 also suppresses EndMT in cardiac ECs directly targeting prss23, a gene that activates Snail signaling, and is essential for CEC proliferation and cardiac vascularization after myocardial infarction [105]. Additionally, miR-155 is upregulated in response to TGF-β and hypoxic conditions and negatively regulates RhoA, an essential player in cell motility, suggesting it functions as a negative feedback mechanism in this process [106].

Recent studies have shown that miRs, particularly miR-200b and miR-18a-5p, are critical mediators of glucose-induced EndMT [107,108]. The miR-200b effect is associated with the upregulation of TGF-β1 and downstream effectors like Snail, Smad2, and p300, a known target of miR-200b [107]. Similarly, miR-18a-5p inhibits EndMT by targeting Notch2 in human aortic valvular ECs. miR-18a-5p levels reduction, thereby increasing Notch2 expression and promoting EndMT. Overexpression of miR-18a-5p suppresses Notch2 and inhibits EndMT, also reducing myocardial fibrosis in diabetic cardiomyopathy models [108].

In contrast to miRs that suppress EndMT, several miRs promote EndMT by targeting molecules that normally inhibit the process. For example, miR-21 is upregulated during TGF-β-induced EndMT in HUVECs. Its inhibition partially prevents EndMT by targeting PTEN, which leads to activation of the Akt pathway and promotes EndMT. In vivo, miR-21 is also increased in cardiac ECs during pressure overload-induced cardiac fibrosis and is reduced upon miR-21 inhibition [109].

TGF-β-induced EndMT involves upregulation of miR-125b, let-7c, let-7g, miR-21, miR-30b, and miR-195, and downregulation of miR-122a, miR-127, miR-196, and miR-375. Among them, miR-125b promotes EndMT by targeting and downregulating p53, a known suppressor of TGF-β-driven fibrotic responses [110]. miR-27b, part of the miR-23/24/27 cluster, acts as a positive regulator of TGF-β-induced EndMT in mouse pancreatic microvascular ECs. It is upregulated by TGF-β1 and promotes EndMT by targeting genes such as Elk1, neuropilin 2, Plexin A2, and Plexin D1 [111].

In a pulmonary hypertension mouse model, miR-130a was found to be upregulated and to promote TGF-β-induced EndMT in lung microvascular ECs. This miRNA is regulated by NF-κB and targets BMPR2, a receptor known to inhibit EndMT [112]. Under hypoxic conditions, miR-126a-5p is upregulated in rat pulmonary microvascular ECs and in a newborn pulmonary hypertension model. This coincides with decreased PECAM-1 and increased α-SMA expression, indicating EndMT. Inhibition of miR-126a-5p reduces this hypoxia-induced EndMT [113].

In particular, in cancer and in fibrosis, context studies have suggested a key role for miR-302c, miR-5703, miR-126-3p, and miR-21-5p.

The overexpression of miR-302c suppresses EndMT, thereby inhibiting hepatocarcinoma tumor growth via the specific inhibition of TGF/TGFR pathway [114].

On the other hand, miR-5703 induces EndMT and disrupts endothelial integrity in tumor-associated ECs in lung cancer via the modulation of ING4 [115]. Additionally, miR-126-3p downregulation in fibrotic disease correlates with endothelial phenotype loss, suggesting its involvement in EndMT inhibition via SPRED [116]. However, in the same context, MiR-21-5p is upregulated in TGF-β2-induced EndMT, and its inhibition restores the endothelial phenotype, suggesting that different miRNAs may cooperate to modulate EndMT via PTEN [109].

RNA deep sequencing under hypoxic conditions identified GATA6-AS as a hypoxia-responsive lncRNA significantly upregulated in ECs. Functional analysis showed that silencing GATA6-AS in HUVECs reduced TGF-β2-induced EndMT, indicating that GATA6-AS is a key regulator of EndMT processes [117]. Furthermore, the lncRNA GATA6-AS inhibits EndMT through its interaction with LOXL2, thereby impeding its H3K4me3 demethylase action and maintaining endothelial gene expression [117]. LINC00961 promotes TGF-β-induced EndMT by suppressing the PTEN/PI3K/AKT signaling pathway [118] and the lncRNA lung adenocarcinoma transcript 1 (MALAT-1) facilitates EndMT through the downregulation of both Smad3 and the TGFBR2-targeting miR-145 [119]. Likewise, the lncRNA H19 prevents EndMT by suppressing TGF-β1 via blockade of the MAPK–ERK1/2 pathway in high-glucose conditions [120]. Conversely, in hypoxic pulmonary hypertension, H19 acts as a promoter of EndMT by upregulating the TGF-β1 receptor TGFβR1 [120]. In pancreas adenocarcinoma, the low expression of LOC340340, LOC101927256, and MNX1-AS1 lncRNAs has been proposed as an EndMT index [121].

Collectively, these studies underscore a complex epigenetic and post-transcriptional regulatory network underlying EndMT. Table 2 summarises the main miRNAs and lncRNAs, their targets, the effects of these on EndMT and the models in which they were studied.

*Radiation.* The role of radiotherapy in cancer has now been established as being dual. While it is commonly used as a therapy to kill cancer cells, it can also induce tissue damage and promote tissue fibrosis by activating chronic inflammation and fibroblasts, as well as stimulating processes such as EndMT [13,20,45,46,47]. Preclinical studies based on colon, rectal, and lung carcinoma tissue models demonstrated that radiation induces fibrosis and remodulation of angiogenesis in ECs. ECs that have been irradiated, in particular, acquire a proinflammatory, procoagulant, and prothrombotic phenotype [13,20,45,46,47]. From a molecular perspective, radiation leads to EndMT in ECs via the induction of DNA damage, ROS production and release, alteration of lipid metabolism, and matrix deposition. Moreover, irradiated ECs are characterized by increased Snail and vimentin expression, as well as downregulation of CD31 synthesis [122]. The Notch signaling pathway and p53 expression are also involved. EC-specific p53 knockout in lung adenocarcinoma-bearing mice and Notch-1 inhibition in neuroblastoma-bearing mice reduced the co-localization of α-SMA and CD31/endomucin, which was increased by irradiation [13,45]. In the context of radiation-induced pulmonary fibrosis (RIPF), radiation induces ndMT in human pulmonary artery ECs via the activation of TGF-βR1/Smad/HIF-1α signaling [123]. Consequently, an increase in mesenchymal cells is evident in the lung parenchyma, characterized by extensive co-localization of α-SMA and CD31 markers, accompanied by exaggerated collagen accumulation [123]. Similar consequences are observed in rectal tissues following radiation treatment [124].

This fibrotic response can create a tumor-supportive microenvironment, potentially resulting in therapy resistance and cancer progression.

In pancreatic and melanoma cancer models, radiation-induced-EndMT leads to resistance to chemo-, radio-, and anti-angiogenic therapies via an abnormal generation and recruitment of pericytes that cover the tumor vasculature [13,47]. In colorectal tumors, radiation-induced EndMT confers resistance to radiotherapy and promotes cancer cell stemness and TAM polarization [13].

In summary, the intricate interplay of diverse signaling cascades, transcription factors, non-coding RNAs, and microenvironmental stimuli underscores the profound molecular complexity driving EndMT.

## 4. Models to Study EndMT

**Figure 3:**
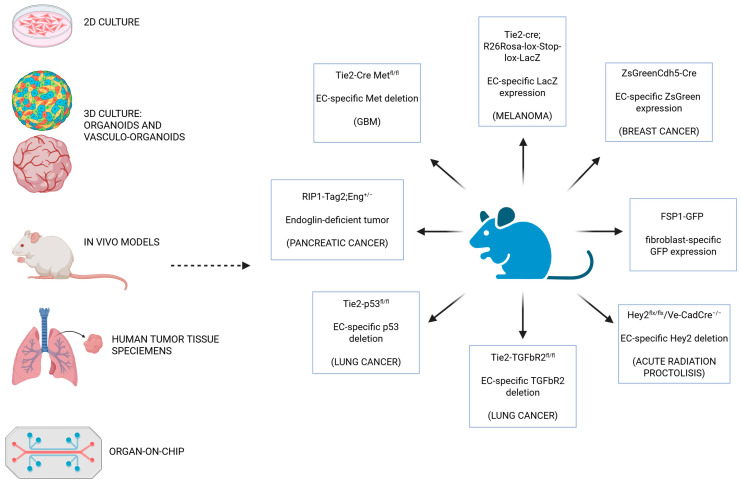
**Overview of experimental models used to study EndMT.** The figure illustrates the main in vitro and in vivo models employed to investigate the mechanisms of EndMT. In vitro approaches include EC cultures stimulated with TGF-β, inflammatory cytokines, or high glucose to induce EndMT. In vivo models include transgenic mouse lines with endothelial-specific lineage tracing and tumor models that reflect pathophysiological conditions where EndMT plays a role. Recently complex models such as organ-on-chip are developed to better replicate the physiological context of EndMT. Created with BioRender.com.

A thorough investigation was performed using advanced 2D and 3D models and in vivo experimental systems to elucidate the molecular mechanisms governing EndMT. The aim of this paragraph is to provide a description of the most commonly used models in the various studies also shown in Figure 3.

*EndMT* in vitro *models.* Studies based on in vitro 2D models provide valuable information about the molecular mechanisms behind EndMT. The experimental conditions, including the tissue of origin of ECs, the choice of the inducing agents as well as the time of induction, are fundamental for the characterization of the multiple sequential and progressive signaling pathways involved. The main EC models investigated include immortalized dermal microvascular ECs (HMEC-1) [125], HUVECs [65,126,127], human esophageal microvascular ECs (HEMECs) [18], tumor ECs isolated from prostate cancer [128], tumor-associated ECs (TECs) from mouse xenografted tumor tissue [129], and human patient tumor biopsies [20]. Moreover, human brain microvascular ECs (HBMECs) were employed to understand the role of EndMT in the formation of brain metastasis in NSCLC [48]. Similarly, human lung microvascular ECs (HMVEC-L) were investigated to define the molecular mechanisms of NSCLC-induced EndMT and their correlation with metastasis [64]. On the other hand, the induction of vessel permeability and transendothelial migration by melanoma and breast cancer cells were analyzed via a primary rat brain EC model [62]. The nature of the matrix for the EC culture is quite important, indeed mesenchymal transition is evident in the presence of a fibronectin-enriched matrix, but not with a collagen or gelatin one [130]. In these conditions, ECs lose their specific endothelial markers and acquire a mesenchymal phenotype. While cell proliferation is maintained, ECs are no longer able to sustain “tubulogenesis” in a Matrigel matrix and they assume a more migratory and invasive phenotype [18].

Short-term stimulation (up to 10 days) induces a reversible EndMT, whereas prolonged stimulation (20 days or more) results in a stable mesenchymal phenotype [29,131]. Recently, thanks to proteomic profiling and single-cell transcriptomic analysis, it has been possible to study the phenotypic plasticity and heterogeneity of ECs during EndMT [132,133]. Some studies suggest a specular process with respect to EndMT, but this remains limited in cancer. In pancreatic cancer, for example, it has been proposed that CAFs can transdifferentiate into ECs via mesenchymal endothelial transition (MendT), thereby contributing to tumor angiogenesis and progression [134].

*EndMT* In vivo *models.* Transgenic mice, based on the modulation of specific EC promoters (*Tie*-2 *or Cdh*5), are used as in vivo models of EndMT to trace the cell-line transition [12,14,32,135]. These models rely on the Cre-LoxP recombination system, in which the Cre recombinase enzyme is expressed under the control of an endothelial-specific promoter. Cre recombinase recognizes specific DNA sequences called loxP sites and catalyzes the excision of the DNA segment located between them. This enables targeted genetic modifications in cells where Cre recombinase is expressed. The Tie2-Cre model is active primarily in ECs and allows for the deletion or activation of genes in the vasculature specifically during early embryonic development. Alternatively, the Cdh5-Cre model offers more restricted endothelial expression and is often preferred when greater specificity is required. Notably, the Cdh5 promoter is employed in tamoxifen-inducible systems (Cdh5-CreERT2), enabling temporal control of gene recombination. This makes it possible to modulate gene expression at specific developmental stages or during disease progression, for example in cancer or fibrosis.

The Tie2-Cre, R26Rosa-Lox-Stop-Lox-LacZ mouse model is a powerful genetic tool for tracing the lineage of ECs [52]. This system combines two key genetic components to achieve cell-specific and heritable gene activation or inactivation. The first is the previously described Tie2-Cre transgene and the second is the R26Rosa-Lox-Stop-Lox-LacZ reporter allele, which is commonly inserted into the Rosa26 locus, a region of the genome known for its ubiquitous expression. In this construct, a “stop” cassette sits between the Rosa26 promoter and the LacZ gene, which encodes the β-galactosidase enzyme, and is flanked by two loxP sites. In the absence of Cre, the stop sequence prevents LacZ expression. In contrast, when Tie2-Cre is expressed in ECs, it recognizes the loxP sites and excises the stop cassette, thereby permanently activating LacZ expression in those cells and marking them. This activation is heritable, meaning all progeny of those cells will also express LacZ, even if they no longer express Tie2 themselves. As a result, researchers can use this model to trace the fate of Tie2-expressing ECs over time by staining for β-galactosidase activity. This model is useful for confirming EndoMT in disease models: if LacZ+ cells are later found to be co-expressing mesenchymal markers such as α-SMA, they have probably originated from ECs.

Via this strategy, in melanoma-bearing mouse models, the role of EndMT in the development of CAFs can be evaluated. In particular, some CAFs co-express the specific endothelial marker CD31 as well as the mesenchymal markers FSP1, or α-SMA. Specifically, for the considered fibrotic regions, 30% of CAFs are lacZ+, 40% of CAFs are FSP1+ CD31+, and 11% of CAFs are α-SMA+, among which 12% were lacZ+. The LacZ model is also implemented to examine the role of bleomycin in lung fibrosis where it is shown that, after bleomycin treatment, the lung fibrotic areas are characterized by an enrichment of LacZ-positive fibroblasts deriving from ECs [135].

Another useful mouse model for tracing EndMT is the ZsGreenCdh5-Cre reporter mouse model [29]. This transgenic system is designed to specifically label and trace ECs using a fluorescent reporter. The ZsGreen reporter gene is usually inserted into the ubiquitous Rosa26 locus and is preceded by a transcriptional stop cassette flanked by loxP sites, which prevent the expression of ZsGreen. When Cre recombinase is expressed in Cdh5-positive cells, it removes the stop cassette via recombination at the loxP sites, which permanently activates the expression of ZsGreen, a bright green fluorescent protein. As a result, these ECs and their descendants (such as CAFs) permanently express ZsGreen and can be easily visualized using fluorescence microscopy or flow cytometry. These mice models confirmed that only a fraction of vessels in breast and K-RasG12D lung tumors contain α-SMA+ ECs, suggesting that EndMT does not involve all ECs after TGF-β stimulation in vivo. The frequency of CD31^+^/α-SMA^+^ ECs could be due to a longer tumor growth period, or to the vascular bed plasticity [29].

c-Met promotes EndoMT and abnormal vascularization, contributing to GBM progression and chemoresistance [12]. To study its in vivo role, researchers generated a mouse with EC-specific c-Met knockout (Tie2-Cre; Met^fl/fl^). Deletion of c-Met in ECs reduces co-expression of FSP-1 and CD31, confirming its role in promoting EndoMT in vivo. Surprisingly, however, deletion of c-Met in ECs does not impair normal angiogenesis, but rather leads to the formation of more normal-looking tumor blood vessels, and reduces hemorrhaging and necrosis, key features of aggressive GBM. Furthermore, endothelial Met knockout significantly improves animal survival after chemotherapy [12].

Endoglin, which is specifically expressed by ECs, is a co-receptor of TGF-β and a key modulator of vasculogenesis, angiogenesis, and inflammation, promoting cell proliferation, migration, and tube formation [136,137,138]. In breast, colon, and lung carcinoma, endoglin is upregulated in ECs, and high expression is associated with poor survival [139]. Consequently, ablation or inhibition of endoglin reduces angiogenesis and tumor growth in cancer mouse models [139]. A well-established RIP1-Tag2 mouse model, which mimics the stepwise progression of pancreatic neuroendocrine tumors, was assessed. Mice deficient for one copy of the endoglin gene (RIP1-Tag2;Eng^+/−^ mice) are characterized by tumor vessel alteration and high metastasis due to a weakened endothelial barrier as a consequence of the increase of EndMT [139].

As previously described, both Trp53 and TGF-β are key regulators of radiation-induced EndMT. To evaluate the molecular mechanisms involved, researchers developed EC-specific Trp53 knockdown and knockout mouse models (Tie2-Cre;Trp53^flox/+^ and Tie2-Cre;Trp53^flox/flox^, respectively), as well as EC-specific Tgfbr2 knockdown mice (Tie2-Cre;Tgfbr2^flox/+^) [13]. Tumor cells isolated from a spontaneous lung adenocarcinoma were implanted in these mice, and analysis of the results suggests that only Trp53 KO inhibits radiation-induced EndMT, thereby supporting tumor regrowth and metastasis, partly due to reduced osteopontin expression. In this context, M2 polarization and recruitment are reduced due to CXCR4/SDF-1 signaling [13].

Hey2 is a Notch target gene involved in physiology and stress-induced EndoMT [124]. To better understand Hey2’s role during irradiation, the transcription factor was inactivated in the endothelial compartment using the Cre-LoxP strategy. Hey2^flx/flx^/Ve-CadCre^+/−^ mice present a reduction in EndMT after irradiation compared to control mice at the level of the intestine and rectum, as well as a mitigation of radiation damage [124].

*Studies using human tumor tissue specimens.* To investigate the relevance of EndMT in human cancers, various studies have analyzed human tumor tissue specimens to evaluate the expression of specific EndMT markers. The resulting data provided important insights into the presence and extent of EndMT within neoplastic tissues. This contributes to the molecular characterization of tumors and could potentially guide the development of targeted therapeutic strategies. Human biopsies were the starting point for studying the role of osteopontin (OPN) in inducing EndMT in colorectal cancer (CRC) [22]. EndoMT-derived cells that co-express α-SMA and CD31 were found in close proximity to macrophages and cells that express OPN. Treatment of HUVECs with OPN was found to decrease the levels of VE-cadherin, Tie1, Tie2, and CD31 mRNA and protein, while increasing the levels of α-SMA and fibronectin. This treatment was also found to reduce cell–cell junction integrity and enhance cell migration and invasion [22].

A molecular EndoMT index based on the lncRNAs LOC340340, LOC101927256, and MNX1-AS1 was identified in cancer tissue from patients with PDAC. High levels of these lncRNAs are significantly associated with increased mRNA levels of both α-SMA and CD31, as well as advanced T4 tumor staging and a high level of M2 macrophages in close proximity to EndoMT cells [121].

In order to evaluate the clinical significance of radiotherapy in the induction of EndMT in lung cancer, researchers examined lung tissue samples from patients who had undergone irradiation and those who had not. EndMT associated with tumors was observed more frequently in lung cancer tissues from patients who underwent irradiation. These tumors are also characterized by the presence of abundant OPN+CD44v6+ cancer stem cells and SDF-1+CD206+ macrophages [13]. Studies on biopsies were also performed to explore the role of EC plasticity in GBM chemoresistance. The results of these studies suggest an EC transformation into stem-like cells, mediated by a c-Met/β-catenin/MRP-1–dependent mechanism that sustains tumor treatment resistance. [13]. Studies based on esophageal adenocarcinoma (EAC) tissues confirm the induction of EndMT by the proximity of ECs and cancer cells and by the TGF-β2 and IL-1β pathway. EACs are characterized by an increase in CAFs that co-express EndMT markers such as CD31 and FSP1, which are found away from blood vessels and near the tumor’s invasive front. These cells also express high levels of VEGF and low levels of VEGFR, as confirmed by co-localization with endothelial and fibroblast markers [18].

*Chip models.* The need to replicate the processes underlying tumor development, growth, and progression in an increasingly physiological way has led researchers to develop increasingly complex 3D models [140]. These systems are certainly based on the ability to replicate the interaction between different cell types (tumor, stroma, and immune cells), as well as the vascular component necessary for sustenance. To this end, tools such as microfluidic systems have been developed. These systems allow ECs to self-assemble into lumenized microvessels within an extracellular matrix under controlled fluid flow. These platforms replicate shear stress and support perfusable networks, enabling real-time visualization of angiogenesis, barrier function, and tumor cell interactions, including intravasation and immune cell infiltration. These 3D models bridge the gap between traditional 2D cultures and animal studies, providing modular, physiologically relevant tools for exploring vascular plasticity, EndoMT, and potential therapeutic interventions in cancer and other vascular diseases. Technological advances in this field are evolving rapidly, to the extent that the operating conditions of these systems are often determined by the tumor model rather than the pathological or physiological analysis of interest. For this reason, we refer to specialized reviews on the subject for a more detailed presentation of the model [140]. In particular, via micro-fluidic devices technologies, different studies evaluated the role of melanoma, lung cancer, and breast cancer exomes in the induction of EndMT [141,142]. Focusing on EndMT, some studies were performed with Organ-on-chip (OoC) platforms that incorporate live cells, tissues, and extracellular matrix within precisely engineered microstructures, able to recreate “vessel flux” and essential aspects of organ architecture and function, and physical and biochemical stimuli. In particular, the induction of EndMT by TGF-β and TNF-α was studied using a liver-on-chip model based on liver-specific endothelium and hepatocytes [143]. In the study was the development of a novel fluorescent-inducible EndMT reporter, the CNN1-eGFP construct, which enabled the identification, tracking, and characterization of the the migratory behavior of EndMT cells. Following TGF-β and TNF-α treatment, live-cell microscopy revealed a significant increase in the number of cells undergoing EndMT compared to the control group. Furthermore, these cells exhibited a higher migration velocity than untreated ECs [143]. A cystic fibrosis (CF) lung tissue model was developed by combining primary human bronchial epithelial cells grown at an air–liquid interface with primary human lung microvascular ECs. This model successfully mimicked the infiltration of polymorphonuclear leukocytes (PMNs) observed in cystic fibrosis (CysF) patients, which is a key contributor to fibrosis progression [144]. Additionally, a three-channel microfluidic device was used to model sprouting angiogenesis in systemic sclerosis (SSc). TGF-β and TNF-α reduced sprout formation, thereby replicating vasculopathy. Human serum from SSc patients exhibited similar, albeit weaker, effects, likely due to the patients’ ongoing treatment with anti-inflammatory and antifibrotic therapies [145].

*Meta-analysis study*. A meta-analysis study, based on TGCA datasets corresponding to different cancer types, was recently conducted to examine changes in EndMT gene expression and its impact on tumor patient prognosis [146]. Breast cancer is the most represented cancer in the distribution of studies. Gene expression analysis of various EndMT markers confirms the characteristic downregulation of PECAM1 during EndMT. However, significant variability in the expression levels of many EndMT-related genes is also observed within specific tumors and between different tumor types. The genes that most characterize EndMT in different tumor types are PECAM1, VWF, CD34, CDH5, MCAM, and CLDN5/11. Analysis of the correlation between the expression of these genes and patient survival shows that reduced expression of MCAM and CLDN11 is associated with poorer overall and relapse-free survival in breast cancer patients. Downregulation of PECAM1, VWF, CHD5, and CLDN5-11 correlates with poor overall survival and progression in lung cancer. As the authors of the meta-analysis suggested, the level of expression detected could be influenced by various factors, such as the tumor’s stage of progression at the time of sample collection, the patient’s genetic and epigenetic factors, and the specific anti-cancer regimens.

## 5. The Influence of EndMT on Tumor Progression

EndoMT is increasingly recognized as a fundamental process involved in multiple stages of tumor progression. By promoting EC plasticity and vascular remodeling, EndoMT contributes to tumor proliferation by supporting a microenvironment that favors rapid cancer cell growth and metastasis. Furthermore, EndoMT influences the tumor’s ability to evade immune surveillance by modulating immune cell infiltration and promoting immunosuppressive conditions within the tumor microenvironment. For these reasons, EndoMT is a critical driver of both metastatic dissemination and tumor expansion and immune escape, making it a promising target for comprehensive anti-cancer therapies. Recent studies have also shown that EndoMT plays a pivotal role in forming the pre-metastatic niche. Tumor-derived signals, such as cytokines, extracellular vesicles, and growth factors, can activate EndoMT at distant sites. At metastatic sites, EndMT inducers lead to increased vascular permeability, stromal remodeling, and immuno-modulation, making these sites more hospitable to circulating tumor cells and facilitating successful colonization. Therefore, EndoMT is a key driver of both local tumor dissemination and systemic metastatic spread through its contribution to pre-metastatic niche formation [4,5,6,7].

We will analyze the impact of EndMT at different stages of tumor progression below.

*EndMT and tumor growth.* As we know, ECs play a central role in shaping the tumor vascular niche. This niche supplies oxygen and nutrients to support tumor growth and creates a protective environment. ECs secrete various angiocrine factors, such as VEGF, IL-6, FGF2, and angiopoietin, which influence cancer cell proliferation, stemness, immune modulation, and resistance to apoptosis. EndoMT also supports tumor cell proliferation by activating ECs to secrete factors that directly stimulate cancer growth [4,5,6,7]. EndoMT-derived ECs release proteins such as eHSP90α, which enhance the proliferation, invasiveness, and tumorigenicity of colorectal cancer cells [22]. A transient EndoMT-tumor niche also promotes tumor cell proliferation in hepatocellular carcinoma [17] and breast cancer [19]. However, further studies are necessary to better understand the specific mechanisms.

*EndMT, angiogenesis, and vessel remodeling.* Pathological angiogenesis is one of the most important mechanisms that sustains tumor growth. Interestingly, much evidence underlines a similarity of this process with EndMT so much so as to indicate the process of angiogenesis as a partial EndMT [147]. In particular, endothelial tip cells, similarly to ECs which make the transition to the mesenchymal phenotype, are able to remodulate their apical–basal polarity, weaken their cell–cell contact, and degrade the extracellular matrix [148]. These cells express high levels of Slug and Snail transcription factors, which, as previously described, are two of the most important transcription factors involved in EndMT [147]. Interestingly, the inhibition of Slug expression in an ovarian carcinoma model significantly decreases angiogenesis and tumor growth [149]. Extracellular vesicles (EVs) secreted from colon cancer cells, WNT5B produced by oral squamous carcinoma cells, and VEGF-TGF-β pathway expression sustain partial “EndMT” angiogenesis [150,151]. As a consequence of a prolonged induction, some tip ECs may maintain the mesenchymal phenotype indefinitely and become “mural cells” as pericytes or vascular smooth muscle cells, therefore stabilizing the neovasculature during vasculogenesis and angiogenesis [152].

EndMT also plays a role in vascular remodeling. During EndMT, via the EC cytoskeletal remodeling, the reduction of the expression of adhesion molecules, the profound changes in endothelial junctional protein expression, and the alteration of signaling pathways in the endothelial barrier permeability increase leading to extravasation and migration of cancer cells [153]. Additionally, EndMT induction by melanoma cancer cell conditioned medium resulted in the decrease in transendothelial electrical resistance, increased adhesion between cancer metastatic cells and ECs, as well as the transendothelial migration of cancer cells [153]. Furthermore, during EndMT, an abnormal recruitment and development of pericytes is also demonstrated in lung cancer [13] and melanoma [154]. Pericytes cover the vessels and could entail resistance to chemotherapy and antiangiogenic therapy [154,155].

*EndMT and cancer-associated fibroblasts (CAFs).* CAFs are one of the most important stromal components of the tumor microenvironment (TME). They can arise from various cell types, such as resident fibroblasts in the TME, as well as via the transdifferentiation of different cells, including bone marrow-derived precursors or mesenchymal stem cells from the bone marrow, vascular smooth muscle cells, pericytes, epithelial cells, adipocytes, and cancer cells through epithelial-to-mesenchymal transition (EMT) [156]. Additionally, ECs through EndMT lead to the development of myofibroblastic/mesenchymal cells, which represent a unique source of CAFs [14]. As previously described, in glioma and in melanoma, the percentage of CAFs originating from EndMT is 50% and 40%, respectively [12,14]. These cells can regulate tumor growth by releasing tumor growth factors and cytokines, and they are also involved in TME remodeling, metastasis, immunosuppression, and drug resistance [63,157]. The CAF secretome comprises TGF, EGF, FGF, VEGF, and matrix metalloproteinase (MMP), which strongly support tumor progression and invasion, as well as angiogenesis and driving monocyte M2 polarization [63,156,157,158]. Parallelly, CAFs are able to modulate tumor EC activity and vessel remodeling sustaining a pro-inflammatory environment via the elevated expression of TNF-α, TGF-β1, IFN-γ, IL1-β, and MCP1. For these reasons, the EndMT process has been linked to the progression of pancreatic, melanoma, and lung cancers as well as indirectly to the increased fibrosis of the surrounding stroma [14,63,156,157,158].

*EndMT and immune modulation.* For their anatomic location, ECs are the first cells that interact with pathogens and stimuli in the bloodstream, supporting directly or indirectly the immune system activation [159,160,161]. In addition to their physiological functions, ECs are involved in cancer progression: they contribute to shaping the tumor microenvironment also by modulating leukocyte adhesion, activation, and transmigration [159,160,161,162]. EC molecular alterations, related to tumor vessel remodeling and tumor EndMT process development, lead to an immune-hostile microenvironment. Conversely, the immune system also regulates EndMT. In particular, as previously described, EndMT leads to the reduced expression of adhesion molecules like ICAM-1 and VCAM-1 which are crucial for T-cell adhesion to blood vessel walls and for their extravasation. During EndMT, ECs and stroma cells via the synthesis of cytokines and growth factors are able to promote the development of TAMs and to impair the activation and survival of T cells [159,160,161]. In lung cancer, alveolar capillary ECs express C–C motif chemokine receptor-like 2 (CCRL2) and the loss of this nonredundant regulator of Natural killer (NK)-cell homing leads to impaired NK cell infiltration, promoting tumor growth [163]. In melanoma, ECs overexpress retinoic acid’s early inducible gene-1e (RAE-1e), a ligand of NKG2D receptor, promoting NKG2D internalization and causing NK desensitization [164]. In parallel, neutrophils, via the synthesis of extracellular traps (NETs) [165,166], promote EndMT: ECs express the NET-DNA receptor CCDC25 and upon engagement, the angiogenic stimulus is enhanced, promoting cell proliferation and the increase in microvessel density [167]. ECs may create an immunosuppressive TME also by upregulating PD-L1 and promoting T Regulatory cells’ (Tregs) activation [168]. B7 homologous 3 (B7H3), an immune checkpoint molecule associated with poor prognosis, tumor progression, and immune suppression, is found to be upregulated in TECs and to dampen T-cell response, highlighting its potential role in regulating the vasculature and the immune evasion in TME [169,170].

ECs engage interactions with adaptive immunity within tertiary lymphoid structures (TLS), expressing ICAM-1, VCAM-1, and producing chemokines such as CXCL13 to recruit and retain B cells. In parallel, B cells via the STAT3 pathway induce angiogenesis and vessel remodeling [171,172]. In hepatocellular carcinoma, ECs promote CD8^+^ T-cell exhaustion through the upregulation of glycoprotein nonmetastatic melanoma protein B (GPNMB) [173]. Additionally, the macrophage migration inhibitory factor (MIF)–CD74 axis is enriched between ECs and immune cells, promoting TAM recruiting or T-cell suppression and leading to a more immunosuppressive environment [174]. On the other hand, recent findings support that CD163+ macrophages play an active role in promoting EndMT, thanks to the secretion of inflammatory cytokines such as IL-1β, TNF-a, C-C chemokine ligand 5 (CCL5), and monocyte chemoattractant protein (MCP-1) [175,176].

Radiotherapy-induced EndMT enhanced TAM proliferation and polarization toward M2-like macrophages. M0 macrophages in co-culture with radiation-EndMT-derived tumor ECs differentiate in Arg1^+^ M2 cells. In contrast the inhibition of irradiation effects in ECs via p53-KO supports the polarization of the macrophages in iNOS^+^ M1 subtype. In vivo, a mild irradiation of tumors led to the recruitment of monocytes and F4/80^+^ macrophages, while a strong irradiation increased the quantity of M2 macrophages in wild-type tumors that were inhibited in EC-p53KO one [13]. In PDAC tissue, EndMT significantly correlates with the enrichment of the M2-macrophage cell population: in vivo and in vitro studies suggested that the EndMT-M2 polarization is mediated by the endothelial expression of Heat shock protein-90-alpha (Hsp90-α) [121].

To better understand the molecular mechanisms underlying EndMT, a transcriptomic analysis study was performed on HUVECs, arterial ECs, and HMECs treated with a cytokine combination of TGF-β1 and IL-1β [177]. Focusing on the EndMT immuno-modulation field, gene set enrichment analysis (GSEA) on the differentially expressed genes after cytokine treatment showed an enrichment of the “chemokine signaling pathway” and of “chemokine receptors bind chemokines” categories. In particular, G0 analysis highlighted that ECs upregulate immune-related processes such as leukocyte activation, migration, and cytokine-mediated signaling pathways. Based on the above-mentioned G0 analysis, several upregulated chemokine-encoding gene classes were identified, including CC chemokines (*CCL2*, *CCL20*, *CCL3L1*, *CCL5*, *CCL7*, and *CCL8*), CXC chemokine (*CX3CL1*), and CX3C chemokines (*CXCL1*, *CXCL2*, *CXCL3*, *CXCL5*, *CXCL6*, *CXCL8*, *CXCL10*, *CXCL11*, and *CXCL12*). Cytokines and growth factor gene classes are represented by *CSF1*, *EDN1*, *HBEGF*, *IL1B*, *IL6*, *IL12A*, *PDGFB*, *PDGFRA*, *TGFB2*, *VEGFA*, and *VEGFC* [177]. An additional way of EndMT regulation of tumor-infiltrating lymphocytes can be assumed by the transformation of ECs in CAFs, the altered angiogenesis and vessel perfusion and permeability [178].

*EndMT and metastasis.* The metastatic process is a multi-step system which includes infiltration of tumor cells in adjacent tissues, followed by intravasation into the bloodstream, survival in the circulation, extravasation, and subsequent proliferation in different organs [179]. In order to colonize distant tissues, some tumor cells activate EMT which causes the loss of their epithelial features and the acquisition of mesenchymal characteristics, enhancing migration and stem-like properties [179]. The deep reorganization of ECs caused by EndMT may lead to an easier intra-/extravasation of cancer cells, facilitating the metastatic process. This hypothesis has been confirmed by different works, suggesting the importance of EndMT in several tumor types, both at the primary tumor site and at the metastatic level [153,179].

In NSCLC, the Rho/RACK pathway dysregulation causes the alteration of EC actin stress fibers and tight junctions, leading to vessel hyperpermeability and a higher metastatic rate [180,181]. In breast and colon-rectal cancer [19,40], EndMT promotes the metastasis of breast cancer cells and colon cancer cells by signaling modulation activated by cell-to-cell contacts or via the secretion of HSP90-α [121]. Studies of early-stage metastasis in an orthotopic murine breast cancer model suggest an increase in permeability of pulmonary endothelium [129]. In melanoma, cancer cells induce EndMT in brain endothelial cells, weakening the barrier integrity. In doing so, tumor cells increase the adhesion between endothelial and melanoma cells, favoring their transendothelial migration [62].

Additionally, several studies have underlined the role of extracellular vesicles, released by breast cancer or OSCC cells, in the mesenchymal transition of liver sinusoidal ECs [65]. Moreover, EVs secreted from Epstein–Barr virus-positive nasopharyngeal carcinoma carry high-mobility-group AT-hook 2 (HMGA2) proteins, and stimulate EndMT and vascular endothelial barrier inhibition, therefore promoting metastasis [182].

EC-endoglin deficiency gives rise to EndMT and favors the metastasis of pancreatic cancer cells to the liver and lungs [139]. Loss of endoglin in RIP-Tag2 mice results in a weak expression of CD31 concomitant with an increase in α-SMA, driving EndMT. Moreover, endoglin deficiency leads to an increased number of metastases due to a feeble endothelial barrier [139]. Furthermore, the inducible deletion of pulmonary EC-specific Adrenomedullin (AM)-RAMP2 signaling results in increased permeability of tumor vessels, enhancing tumor metastasis [183]. A1AT expression is higher in lung cancer tissue compared to the healthy one. Specifically, higher levels of A1AT promote both EndMT and migration of cancer cells. Its overexpression also enhances mesenchymal markers, such as vimentin and N-cadherin, while its downregulation significantly inhibits EndMT and lung cancer metastasis [64]. PLEK2 is another important element in NSCLC, which promotes EMT, metastasis, and EndMT, downregulating tight junctions and suppressing ECs’ barrier function. PLEK2 overexpression causes a reduction of endothelial markers, impairs the expression of tight junction proteins, such as ZO-1 and occludin, and increases mesenchymal markers. In contrast, its knockdown inhibits the TGF-β1-induced EndMT and the disruption of the vascular barrier [64].

While considerable focus has been given to EndoMT at primary tumor sites, much less attention has been paid to its role in metastatic locations. EndoMT has not yet been clearly observed in human metastatic tumor tissues, and further studies are needed to determine whether it occurs before or after metastatic spread in human cancers. In a mouse model of breast cancer, EndoMT in the lung, along with increased vascular permeability, is significant during the early stages of metastasis [133]. Similarly, EndoMT in brain ECs, and the consequent weakening of the barrier, is evident in melanoma [65]. In breast cancer, extracellular vesicles can induce EndoMT in liver sinusoidal ECs (LSECs) [183]. These findings imply that tumor cells can actively drive EndoMT at metastatic sites, helping to establish a supportive pre-metastatic niche and facilitating the escape of tumor cells from blood vessels into tissues, thus promoting metastasis.

**Figure 4:**
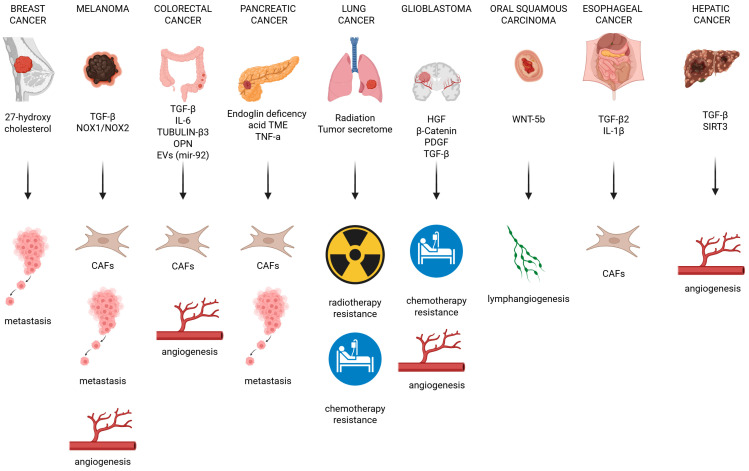
**Representation of tumor-specific EndMT regulators and their functional impact across different cancer types.** The figure illustrates various tumor types (e.g., breast, lung, pancreatic, and colorectal cancers) alongside key molecular regulators of EndMT. For each cancer type, the associated EndMT drivers are shown, together with the downstream effects of EndMT, such as enhanced tumor fibrosis, immune evasion, angiogenesis modulation, and metastasis promotion. Created with BioRender.com.

In summary (Figure 4), the effects of EndMT are mediated by distinct molecular regulators across different tumor types, and its final outcome can vary depending on the specific tumor context, highlighting the complexity and heterogeneity of the EndMT process in cancer.

## 6. EndMT and Treatment Resistance

Despite significant improvements in oncological clinical care through the introduction of new targeted therapies, acquired drug resistance is confirmed to be one of the main reasons related to poor prognosis in lung cancer. Resistance can be achieved by the acquisition of new mutations in the driver oncogenes, the activation of alternative signaling pathways, or the histological transformation towards a new cancerous phenotype [184]. Recent discoveries highlight how EndMT can also act as an alternative resistance mechanism to cancer treatment. Indeed, standard cancer therapies, i.e., radiotherapy and chemotherapy, appear to induce EndMT in several types of neoplasia [13,45,124,184,185] and its inhibition can restore treatment sensitivity [12,21]. Actually, the stromal compartment of the tumoral niche, following chemotherapy, seems to be partially responsible for the survival of resistant residual cancer cells [186]. As previously mentioned, CAFs can be developed through EndMT and, specifically, the CD10- and GPR77-positive CAF subset exhibits chemoresistance in lung cancer patients [187]. Additionally, CAFs interfere with the platinum response in cancer cells and facilitate tumor growth and progression. By secreting oncogenic signals and angiogenic-inducing factors, such as TGF-β and VEGF, respectively, CAFs are able to promote cancer invasion and angiogenesis [5,188,189]. In particular, high VEGF expression correlates with platinum resistance and its suppression is able to restore sensitivity to chemotherapy in lung cancer [5,190]. Studies on GBM tissues identify a HGF/c-Met-mediated EndMT process as a chemoresistance mechanism to temozolomide [12]. More specifically, temozolomide and anti-VEGF therapy resistance is linked to the high expression of drug pumping and efflux proteins’ ABBC1/Multidrug resistance associated protein (MRP-1) [71] and to the VEGFR2 protein level reduction sustained by EndMT [37].

Furthermore, within the TME, an abundance of EndMT signature is associated with a worst outcome and correlates with cisplatin resistance [188]. As a matter of fact, EndMT is found to be one of the several biological responses after radiation therapy in Human Lung Microvascular Endothelial Cells (HLMVEC) [46]. As stated above, radiation-induced EndMT results in the polarization of TAMs towards an M2-like phenotype and the formation of an abnormal vasculature. This set of events proves to be responsible for tumor regrowth and progression after radiotherapy [13]. A reciprocal crosstalk between cancer cells and the TME seems to be crucial in the acquisition of a drug-resistant phenotype. Indeed, resistant NSCLC cells induce EndMT in HUVEC cells in a multicellular tumor spheroid model through the activation of GSK-3β. Interestingly, by inhibiting GSK-3β, sensitivity to Gefitinib and Cisplatin is restored [65].

Similar results were reported with CHIR-99021, a GSK-3β inhibitor, which is able to re-establish susceptibility to radiation by EndMT suppression [191].

In this context of crosstalk within the tumoral niche, A1AT secreted by cancer cells seems to be a key player: its overexpression reduces cisplatin sensitivity through EndMT in surrounding HMECs [64]. Indeed, cancer secretomes happen to increase EndMT characteristics and angiogenic capability in ECs, contributing to drug resistance [115]. Actually, the angiogenic process induced by EndMT correlates with a worst survival outcome and targeting angiogenesis could be a potential therapeutic approach [188]. It could reduce the number of EndMT-induced CAFs in the TME, improving the overall prognosis for patients exhibiting treatment failure [192,193].

## 7. EndMT Inhibitors: Bench to Bedside Struggles

EndMT contributes to several aggressive and harmful behaviors in tumors, and targeting this process by either inhibiting or reversing it during cancer progression may offer promising therapeutic benefits. In theory, disrupting the signaling pathways and molecules that drive or sustain EndMT, or blocking those that support tumor-promoting activities, could help to slow tumor development. However, caution must be exercised when targeting EndMT due to the high variability of this process. Targeting pathways such as TGFβ could inhibit EndMT and suppress tumor growth. Although TGF-β is a promising candidate for inhibiting EndMT and tumor progression, its broad range of actions and multiple cellular targets make it difficult to control and are lacking in specificity. To minimize off-target effects, anti-EndMT strategies should ideally focus on TEC markers, bearing in mind that EndMT also plays a role in normal physiology [7]. As shown in Figure 4, there is considerable variation in the expression of EndMT markers across and within tumor types, driven by different biological conditions and overlapping molecular processes. Therefore, identifying precise EndMT-related biomarkers is crucial for the development of effective, targeted therapies that are distinct from broader anti-angiogenic treatments, which frequently lack specificity and efficacy. However, only a limited number of studies in the field of cancer have addressed this challenge, with most focusing on assessing the effectiveness of existing drugs. We list some possible specific targets below.

Like vinorelbine, paclitaxel and similar compounds, eribulin acts directly on cell microtubules, specifically blocking their elongation and promoting the formation of non-functional tubulin clusters. This disruption prevents the formation of proper mitotic spindles, resulting in permanent arrest of the cell cycle at the G2-M phase and ultimately triggering apoptosis [194]. Eribulin has been approved by both the EMA and the FDA for the treatment of metastatic breast cancer. Its effects have been studied in clinical trials involving patients with advanced or metastatic breast cancer, as well as in a patient-derived orthotopic xenograft (PDOX) mouse model of primary breast osteosarcoma [195,196,197]. Eribulin has demonstrated the ability to counteract TGFβ-induced EndMT and suppress angiogenesis [194]. Notably, its therapeutic effect appears to be associated with high levels of circulating lymphocytes, suggesting that immune-related mechanisms may contribute to its activity. The pharmacological effects of eribulin may also be mediated through three off-target mechanisms [194]. Firstly, it appears to promote vascular remodeling by increasing the transcription of CD31 and VE-cadherin. Additionally, eribulin may enhance the infiltration of immune cells into the tumor microenvironment by upregulating these same markers along with ICAM-1. Finally, it appears to inhibit EndMT driven by TGF-β. These findings suggest new potential therapeutic avenues and indicate that eribulin could be particularly effective in tumors with an immune-excluded phenotype. Eribulin treatment consequently induces vascular remodeling associated with improved perfusion in breast cancer xenograft models [198].

Similarly, nudifloside (NDF), a compound derived from traditional Chinese medicine, can reverse EndMT triggered by TGFβ-1 by blocking Ezrin phosphorylation [199]. NDF can suppress the increased migration, invasion, and F-actin organization that are typically observed in ECs exposed to TGFβ-1. It reverses the expression of various EndMT-related biomarkers, restoring the typical morphology of ECs and their ability to form tube-like structures, which are altered by TGFβ-1. Furthermore, NDF treatment significantly disrupts VEGF-induced angiogenesis in both in vitro and ex vivo models [199].

Octyl gallate (OG), an ester of 3,4,5-trihydroxybenzoic acid, is used as an antioxidant and preservative in food additives and cosmetics. Furthermore, studies in breast, colon, and lung tumor models in animals or human cancer cell lines suggest that OG has chemopreventive and anti-carcinogenic properties [200]. In a mouse model of pancreatic ductal adenocarcinoma involving EndMT, it has been demonstrated that OG inhibits tumor growth, M2-macrophage recruitment, and serum HSP90α levels [201].

Vincristine (VIN) is a commonly used treatment for advanced colon cancer, as it can block cell division at the metaphase stage by binding to tubulin and disrupting microtubule formation. However, as with many chemotherapeutic agents, it can lead to the development of chemoresistance. In vitro studies suggest that exposure of ECs to conditioned medium obtained from co-cultured colon cancer cells and CAF-like cells treated with vincristine can induce an increase in CAF-like cell transition through the EndMT process [202]. In parallel, treating cancer cells and CAF-like cells with non-steroidal anti-inflammatory drugs (NSAIDs) significantly reduces mortality related to breast, colorectal, and lung cancers, decreases EndMT, inhibits cell elongation, and restores the ability of ECs to form capillaries [202].

NEO212, a temozolomide–perillyl alcohol conjugate, can inhibit EndMT by blocking TGF-β and Notch pathways in a glioblastoma multiforme (GBM) mouse model. A study was performed using an in vivo co-culture of glioma stem cells (GSCs) and brain ECs (BECs), both of which were isolated from human specimens. Early after treatment, NEO212 decreases endothelial transition by reducing the expression of mesenchymal markers, thereby blocking the TGF-β and Notch pathways [66].

Galunisertib is an oral small molecule inhibitor of the TGF-β receptor 1 kinase that specifically downregulates the phosphorylation of SMAD2 and is associated with an increase in T-cell infiltration in tumors [203]. In nasopharyngeal carcinoma (NPC), galunisertib enhances the anti-tumor effects of bevacizumab (BEV), an anti-angiogenic agent, when used in combination. Histological analysis of the tumors shows that the combination therapy improves tumor vascular normalization (TVN), extends its duration, and enhances vascular structure and function, despite a decrease in overall blood vessel density [204].

Icariin, an anti-inflammatory, anti-osteoporotic, and immune compound, attenuates endothelial–mesenchymal transition via H19/miR-148b-3p/ELF5 in ox-LDL-stimulated HUVECs [205].

In a lung multicellular tumor spheroid (MTS) model, the GSK-3β inhibitor CHIR-99021 significantly increases the expression of CD31 and VE-cadherin, while inhibiting the expression of α-SMA and vimentin in HUVEC cells [65]. Concurrently, hematoxylin and eosin (H&E) staining and CD31 immunohistochemistry staining demonstrate that the spheroid size increases following treatment and is characterized by numerous CD31-positive cells. Furthermore, combining CHIR-99021 with gefitinib [206] significantly enhances the sensitivity of NSCLC cells to gefitinib in MTSCs, regardless of epidermal growth factor receptor (EGFR) mutation. Treatment with gefitinib plus CHIR-99021 significantly inhibited xenograft MTSC volume versus MTSC treated with gefitinib alone and attenuated the degree of fibrosis. In tumor tissues from untreated mice or mice treated with gefitinib, CD31 was found to be unevenly distributed across a broad area. In contrast, tumors from mice treated with CHIR-99021, either alone or in combination, show a slight reduction in CD31 expression. Additionally, the number of Ki67-positive tumor cells near vessels is lower in tumors from mice treated with CHIR-99021 or the combination therapy than in tumors from untreated or gefitinib-only treated mice [206].

Remarkably, via an in vitro microfluidic model, it is known that exosomes released by mesenchymal stem cells can counteract EndoMT inhibiting the transcription of TGF-β and vimentin [207].

Different TGF-β inhibitors have been developed. In particular, the small molecule galunisertib (LY2157299) (that blocks the kinase activity of the TGF-β receptor I) seems to be one of the most advanced inhibitor, as demonstrated by two clinical trials for pancreatic cancer (NCT01373164) and hepatocellular carcinoma (NCT01246986). Unfortunately, however, there is no evidence of its role in the regulation of EndM [208].

Monoclonal antibodies targeting TGF-β are currently being treated as promising therapeutic agents and are currently being evaluated in various diseases. Fresolimumab, one such antibody initially designed to treat idiopathic pulmonary fibrosis, has progressed to phase II clinical trials for cancers including renal cell carcinoma, melanoma, and metastatic breast cancer (NCT01401062) [209,210]. Notably, blocking TGF-β with fresolimumab not only aims to inhibit tumor-promoting pathways but enhances anti-tumor immune responses. However, further research is needed to confirm fresolimumab’s impact on EndMT and fully understand its therapeutic potential in this context.

In vitro and in vivo model studies suggest that the knockout of TGF-β and Slug in glioblastoma-associated pericytes is able to block EMT and to disrupt tumor blood vessel formation [50].

Table 3A,B provide examples of clinical studies investigating drugs targeting EndMT (as single agent or, more often, as combination treatment) as a therapeutic strategy in different tumor types and fibrotic disorders, respectively. The two EndMT inhibitors more extensively studied in the clinical setting are the two aforementioned compounds, galunisertib and fresolimumab [50,208,209,210]. However, one intriguing aspect of targeting EndMT in cancer and fibrosis is the possibility of “drug repurposing”, also known as “drug repositioning” and “drug reprofiling [211,212]. Drug repurposing aims at extending the label of already-approved drugs, extending their study and role beyond the primary disease for which they initially received marketing authorization; the process can obviously include off-patent or generic drugs, with specific challenges [213]. This would be most likely not as single therapeutic interventions but by adding EndMT inhibitors as “adjuvants” in other treatment schemes, particularly in the oncological setting, similarly to what has been done with newly developed EndMT-targeting agents (e.g., see the studies reported in Table 3A,B). Indeed, a few EndMT inhibitors are not novel compounds but rather could benefit from drug repurposing processes for study design and, possibly, approval for clinical use. Such examples are represented by agents as losartan, an angiotensin-II receptor blocker, and kallistatin [214,215,216] used in the cardiovascular setting. Therefore, learning from other disease settings, the EndMT-inhibiting properties of specific compounds already approved in the clinic could provide a precious source for the investigation of EndMT-targeting agents to be implemented in oncological and anti-fibrotic diseases treatment schemes.

## 8. Conclusions

**Figure 5:**
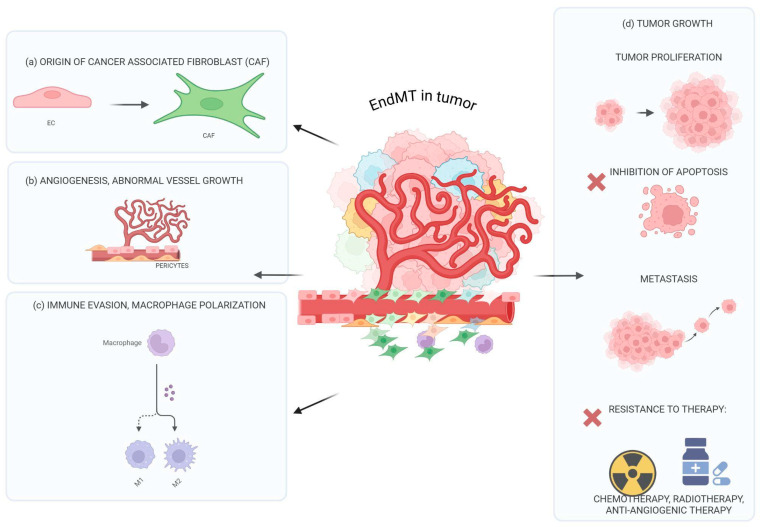
**A schematic illustration of the molecular and cellular mechanisms that characterize EndMT.** During EndMT, ECs lose endothelial markers and gain mesenchymal features. (**a**) This transition contributes to vascular remodeling and enhanced vessel stiffness. (**b**) EndMT-derived mesenchymal cells differentiate into CAFs, which promote tumor progression by remodeling ECM and secreting pro-tumorigenic factors. (**c**) EndMT exerts an immunosuppressive effect by facilitating immune evasion through the expression of immune checkpoint molecules and immunosuppressive cytokines, thereby inhibiting anti-tumor immune responses. The endothelial transition also modulates macrophage polarization, favoring pro-inflammatory or pro-fibrotic phenotypes, and thus shaping the inflammatory microenvironment. (**d**) Furthermore, EndMT promotes therapy resistance by fostering a protective microenvironment that reduces drug penetration and enhances survival signaling pathways in tumor and stromal cells. Created with BioRender.com.

The tumor molecular environment (TME) represents a dynamic and heterogeneous ecosystem where ECs play a pivotal role, not only as structural components of the vasculature but also as active modulators of tumor progression. The growth and survival of cancer cells is strongly linked to the spatial and functional reorganization of stromal cells. EndMT results in the loss of endothelial identity and acquisition of mesenchymal features, which in turn contribute to the rise of CAFs, aberrant vasculature, and fibrotic remodeling. EndMT has a systemic impact promoting tumor heterogeneity, invasion, metastasis, immune evasion, and resistance to chemo-, anti-angiogenic, and immuno-therapy (Figure 5). EndMT does not follow a precise pattern of extra- and intracellular signals, but it is supported by a miscellany of pathways, including, but not limited to, TGF-β, Wnt/β-catenin, and Notch, capable of interacting with each other. Despite numerous studies in this field, the molecular mechanisms underlying EndMT remain mostly unknown, therefore representing an interesting and exciting field of research. Targeting EndMT offers a promising avenue for the development of novel therapeutic strategies aimed at improving oncologic outcomes while simultaneously minimizing treatment-related morbidity. In particular, significant research efforts focus on identifying compounds, chemical agents, and pharmacological substances capable of switching off or reversing EndMT and its related pathological processes. These compounds are designed to modulate the diverse molecular pathways that govern the phenotypic shift of ECs into mesenchymal-like cells, to interfere with the activity or expression of key transcription factors, genes, and epigenetic regulators involved in the transition and, in particular, to inhibit the growth factors that trigger or sustain EndMT. Although these EndMT-targeting therapies can be considered promising, cancer heterogeneity and EndMT processes constitute a significant challenge for drug development. Specifically, a highly selective approach will be necessary to avoid unintended reactions, minimizing on-target, off-tumor effects in healthy tissues, where EndMT also occurs physiologically.

The picture is further complicated by the fact that EndMT is much more dynamic and reversible than previously thought. Instead of being a simple switch from one cell type to another, EndMT happens along a spectrum of different states. Many ECs undergoing this transition do not completely lose their original identity; instead, they adopt hybrid or partial phenotypes, showing features of both endothelial and mesenchymal cells. This reversibility complicates therapeutic approaches significantly. Since the cells can exist in a range of intermediate forms and can switch back and forth, targeting them becomes much harder. Therapies designed to block or reverse EndMT need to consider that cells may not be purely mesenchymal and could quickly adapt or relapse into previous states if the microenvironment changes. This plasticity suggests that treatments must be flexible and possibly target both the cells and the signals in their environment to achieve lasting effects.

In the field of oncology, the shortcomings of conventional in vitro and in vivo models are becoming increasingly evident, particularly when it comes to understanding tumor biology and forecasting therapeutic outcomes. Widely used two-dimensional (2D) cancer cell cultures fall short in replicating the intricate architecture and dynamic microenvironment typical of tumors in the human body. Likewise, although animal models have contributed significantly to cancer research, species-specific differences and ethical challenges limit their effectiveness in translating findings to human contexts. As a result, there is a growing push toward more physiologically accurate models. Among these, tumor-derived organoids have emerged as promising three-dimensional (3D) systems that more closely resemble the structural and functional properties of real tumors. These organoids, often developed from patient tumor samples, maintain key genetic and histological features of the original cancer, making them highly relevant tools for exploring tumor progression, drug testing, and personalized treatment strategies. Nonetheless, conventional organoid cultures are limited by their static nature, they do not reflect the mechanical and fluidic forces present in vivo, such as shear stress, compression, or continuous perfusion. These factors are crucial for accurately modeling tumor behavior, particularly processes like cell migration, invasion, and therapy resistance. To overcome these limitations, scientists have started combining organoid technology with microfluidic platforms, creating advanced tumor organoid-on-chip systems. These microengineered devices allow precise manipulation of the tumor microenvironment, replicating conditions such as blood flow, tissue stiffness, and interactions with surrounding stromal or immune cells. Such elements are known to influence cancer progression and the effectiveness of therapeutic agents. Innovative applications of these models include the recreation of processes like tumor angiogenesis, metastatic spread, and drug diffusion within dense tumor tissues. These platforms are especially useful in studying aggressive and treatment-resistant cancers, such as glioblastoma, pancreatic cancer, and lung cancer, where tumor–microenvironment interactions play a pivotal role in disease dynamics and therapeutic resistance. Ultimately, tumor organoids-on-chip represent a promising frontier in oncological research, in particular, for EndMT studies, offering a high-fidelity, patient-specific approach to studying cancer biology in vitro. By capturing both the structural complexity of tumors and the dynamic cues of their microenvironment, these models hold great potential for improving our understanding of tumor behavior, predicting treatment responses more accurately, and advancing the development of personalized anti-cancer therapies.

Despite an increasing body of experimental evidence supporting the involvement of EndMT in cancer progression, this process is largely overlooked in clinical practice. To the best of our knowledge, the routine assessment of EndMT markers is not currently included in pathological tumor characterization. This lack of clinical integration persists even though EndMT has been implicated in critical aspects of tumor biology, such as fibrosis, immune evasion, and therapy resistance. The absence of standardized protocols for detecting and quantifying EndMT-related markers highlights a significant discrepancy between experimental research and clinical application. Bridging this gap is essential for a full understanding of the clinical implications of EndMT and for realizing its potential as a diagnostic, prognostic, and therapeutic target.

Although the molecular understanding of EndMT has significantly advanced, thanks in part to the development of innovative experimental models, its clinical relevance and potential as a therapeutic target remain limited and require further investigation. Unfortunately, to the best of our knowledge, despite the importance of the process in cancer, there are few clinical trials about EndMT and no therapeutic program for its specific inhibition.

In conclusion, EndMT can be recognized as a pivotal process in tumor progression that can complicate the pathological and treatment picture. For these reasons, any effort to better understand and inhibit EndMT will be critical to improve cancer treatment outcomes.

## Figures and Tables

**Table 1:** **Comparison of EMT and** **EndMT.**

	EMT	EndMT	References
**Cell of Origin**	Epithelial cell	Endothelial cell	[4,5,6,7,8,9,10,11,24,25,26,27]
**Polarity**	Loss of apical–basal polarity	Loss of apical–luminal polarity	
**Cell–Cell Junctions** (loss)	E-cadherin, CD31, vWF, Tie2	VE cadherin, CD31, vWF, Tie2	
**Cell–Cell Junctions** (gained)	N-cadherin, vimentin, fibronectin, collagen type I/III	N-cadherin, vimentin, fibronectin, collagen type I/III	
**Markers**	α-SMA, FSP-1, FAP	α-SMA, FSP-1, FAP	
**Signaling**	Snail, Slug/SNAI2, Twist TGFB1	Snail, Slug/SNAI2, Twist TGFB2 direct, TGFB1 -3 indirect, synergism with inflammatory cytokines (e.g., IL-1β)	
**Endothelial-Specific Regulator**	Sox9 Shear stress	Sox9 ETS transcription factors Shear stress (KLF2)	
**Developmental Role**	Embryogenesis, wound healing	Cardiogenesis, valvulogenesis, vasculogenesis, vascular remodeling	
**Pathological Involvement**	Loss of epithelial traits, pulmonary fibrosis, epithelial cancers, wound healing disorders, metastasis, chemoresistance	Loss of endothelial traits, fibroblast generation, cardiac and renal fibrosis, pulmonary hypertension, tumor microenvironment remodeling, Angiogenesis, metastasis, vascular permeability	

**Table 2:** miR and lncRNA involved in EndMT.

miR	Target	Effect on EndMT	ECs	Ref
**Let-7**	TGF-β	Inhibit	HUAEC, HUVEC	[87]
**miR-200a**	GRB2	Inhibit	HAEC	[98,102]
**miR-20a**	TGF-βR1/2, SARA	Inhibit	HUVEC	[99]
**miR-630**	SLUG	Inhibit	HD-MVEC	[100]
**miR-29**	DPP4	Inhibit	HMVEC	[101]
**miR-23**	Has2	Inhibit	MEEC	[108]
**miR-200b**	Smad, Snail, p300	Inhibit	HRMEC, MHEC	[111]
**miR-18a-5p**	Notch2	Inhibit	HAVEC	[112]
**miR-21**	PTEN	Promote	HUVEC	[113]
**miR-125b**	p53	Promote	MCEC	[114]
**miR-27b**	Elk1, Neuropilin2, PlexinA2, Plexind1	Promote	MS-1	[115]
**mir-130a**	BMPR2	Promote	LMVEC	[116]
**GATA6-AS**	LOXL2	Inhibit	HUVEC	[121]
**LINC00961**	PTEN/PI3K/AKT	Promote	HCMEC	[122]
**MALAT-1**	SMAD3, miR-145	Promote	EPC	[123]
**H19**	MAPK-ERK-1/2 TGFBR1	Inhibit (glucose) Promote (hypoxia)	PAECS	[124,125]

HUAECs: human umbilical artery ECs, HUVECs: human umbilical vein ECs, HAECs: human aortic ECs, HD-MVECs: human dermal microvascular ECs, HMVECs: human dermal microvascular ECs, MEECs: mouse embryonic ECs, HRMECs: human retinal microvascular ECs, MHECs: mouse heart ECs, HAVECs: human aortic valvular ECs, MCECs: mouse cardiac ECs, MS-1s: mouse pancreatic microvascular ECs, LMVECs: lung microvascular ECs.

**Table 3:** **(A)—Clinical** **trials** **targeting** **EndMT** **in** **oncology.** **(B)—Clinical** **trials** **targeting** **EndMT** **in** **tissue** **fibrosis-related** **diseases.**

**(A)**						
**Investigational Agent and Target/** **Mechanism of Action**	**NCT Identifier**	**Title**	**Tumor Type**	**Phase**	**Status**	**Publications**
**Galunisertib** **TGFβRI inhibitor**	NCT01373164	A Study in Metastatic Cancer and Advanced or Metastatic Unresectable Pancreatic Cancer	Pancreatic cancer, advanced or metastatic	I–II	Completed	[217]
	NCT01246986	A Study of LY2157299 in Participants With Hepatocellular Carcinoma	Hepatocellular carcinoma, advanced	II	Completed	[218]
	NCT05700656	Galunisertib Combined With Capecitabine in Advanced CRC With peritoneal metastasis	Advanced colorectal cancer	I–II	Recruiting	Not available
	NCT02452008	Study of TGF-β Receptor Inhibitor Galunisertib (LY2157299) and Enzalutamide in Metastatic Castration-resistant Prostate Cancer	Metastatic castration resistant prostate cancer	II	Active, not recruiting	Not available
**Fresolimumab (GC1008)** **inhibits TGF-β activity by targeting all TGF-β isoforms**	NCT01401062	Fresolimumab and Radiotherapy in Metastatic Breast Cancer	Breast cancer, metastatic	II	Completed	[219]
	NCT01112293	Anti-TGF Monoclonal Antibody (GC1008) in Relapsed Malignant Pleural Mesothelioma	Relapsed malignant pleural mesothelioma	II	Completed	Not available
	NCT02581787	SABR-ATAC: A Trial of TGF-beta Inhibition and Stereotactic Ablative Radiotherapy for Early Stage Non-small Cell Lung Cancer	Non-small cell lung cancer, early stage	I–II	Completed	Not available
	NCT00356460	Safety and Efficacy Study of GC1008 to Treat Renal Cell Carcinoma or Malignant Melanoma	Advanced/metastatic renal cell carcinoma or melanoma	I	Completed	[220]
**(B)**						
**Investigational agent and target/** **mechanism of action**	**NCT identifier**	**Title**	**Disease/** **condition**	**Phase**	**Status**	**Publications**
**Fresolimumab** **inhibits TGF-β activity by targeting all TGF-β isoforms**	NCT01284322	Fresolimumab in systemic sclerosis	Systemic sclerosis	I	Completed	[221]
	NCT01665391	A Study of Fresolimumab in Patients With Steroid-Resistant Primary Focal Segmental Glomerulosclerosis (FSGS)	Focal Segmental Glomerulosclerosis	II	Completed	[222]
	NCT00125385	Study of GC1008 in Patients With Idiopathic Pulmonary Fibrosis (IPF)	Idiopathic pulmonary fibrosis	I	Completed	[223]
	NCT01291784	Anti-TGF-beta Therapy in Patients With Myelofibrosis	Myelofibrosis	I	Completed	[224]

## References

[1] De Visser K.E., Joyce J.A. (2023). The evolving tumor microenvironment: From cancer initiation to metastatic outgrowth. Cancer Cell.

[2] Potente M., Mäkinen T. (2017). Vascular heterogeneity and specialization in development and disease. Nat. Rev. Mol. Cell Biol..

[3] Zeng Q., Mousa M., Nadukkandy A.S., Franssens L., Alnaqbi H., Alshamsi F.Y., Safar H.A., Carmeliet P. (2023). Understanding tumour endothelial cell heterogeneity and function from single-cell omics. Nat. Rev. Cancer.

[4] Potenta S., Zeisberg E., Kalluri R. (2008). The role of endothelial-to-mesenchymal transition in cancer progression. Br. J. Cancer.

[5] Platel V., Faure S., Corre I., Clere N. (2019). Endothelial-to-mesenchymal transition (EndoMT): Roles in tumorigenesis, metastatic extravasation and therapy resistance. J. Oncol..

[6] Yin Z., Wang L. (2023). Endothelial-to-mesenchymal transition in tumour progression and its potential roles in tumour therapy. Ann. Med..

[7] Piera-Velazquez S., Jimenez S.A. (2019). Endothelial to Mesenchymal Transition: Role in Physiology and in the Pathogenesis of Human Diseases. Physiol. Rev..

[8] Dejana E., Hirschi K.K., Simons M. (2017). The molecular basis of endothelial cell plasticity. Nat. Commun..

[9] Ubil E., Duan J., Pillai I.C., Rosa-Garrido M., Wu Y., Bargiacchi F., Lu Y., Stanbouly S., Huang J., Rojas M. (2014). Mesenchymal-endothelial transition contributes to cardiac neovascularization. Nature.

[10] Xiao L., Dudley A.C. (2017). Intro Fine-tuning vascular fate during endothelial-mesenchymal transition. J. Pathol..

[11] Kalluri R., Zeisberg M. (2006). Fibroblasts in cancer. Nat. Rev. Cancer.

[12] Huang M., Liu T., Ma P., Mitteer R.A., Zhang Z., Kim H.J., Yeo E., Zhang D., Cai P., Li C. (2016). c-Met-mediated endothelial plasticity drives aberrant vascularization and chemoresistance in glioblastoma. J. Clin. Investig..

[13] Choi S.H., Kim A.R., Nam J.K., Kim J.M., Kim J.Y., Seo H.R., Lee H.J., Cho J., Lee Y.J. (2018). Tumour-vasculature development via endothelial-tomesenchymal transition after radiotherapy controls CD44v6(+) cancer cell and macrophage polarization. Nat. Commun..

[14] Zeisberg E.M., Potenta L., Xie L., Zeisberg M., Kalluri R. (2007). Discovery of endothelial to mesenchymal transition as a source for carcinoma-associated fibroblasts. Cancer Res..

[15] Gasperini P., Espigol-Frigole G., McCormick P.J., Salvucci O., Maric D., Uldrick T.S., Polizzotto M.N., Yarchoan R., Tosato G. (2012). Kaposi sarcoma herpesvirus promotes endothelial-to-mesenchymal transition through Notch-dependent signaling. Cancer Res..

[16] Garcia J., Sandi M.J., Cordelier P., Binétruy B., Pouysségur J., Iovanna J.L., Tournaire R. (2012). Tie1 deficiency induces endothelial-mesenchymal transition. EMBO Rep..

[17] Zhu K., Pan Q., Jia L.Q., Dai Z., Ke A.W., Zeng H.Y., Tang Z.Y., Fan J., Zhou J. (2014). MiR-302c inhibits tumor growth of hepatocellular carcinoma by suppressing the endothelial-mesenchymal transition of endothelial cells. Sci. Rep..

[18] Nie L., Lyros O., Medda R., Jovanovic N., Schmidt J.L., Otterson M.F., Johnson C.P., Behmaram B., Shaker R., Rafiee P. (2014). Endothelial-mesenchymal transition in normal human esophageal endothelial cells cocultured with esophageal adenocarcinoma cells: Role of IL-1b and TGF-b2. Am. J. Physiol. Cell Physiol..

[19] Ghiabi P., Jiang J., Pasquier J., Maleki M., Abu-Kaoud N., Halabi N., Guerrouahen B.S., Rafii S., Rafii A. (2015). Breast cancer cells promote a notch-dependent mesenchymal phenotype in endothelial cells participating to a pro-tumoral niche. J. Transl. Med..

[20] Choi S.H., Nam J.K., Kim B.Y., Jang J., Jin Y.B., Lee H.J., Park S., Ji Y.H., Cho J., Lee Y.J. (2016). HSPB1 inhibits the endothelial-to-mesenchymal transition to suppress pulmonary fibrosis and lung tumourigenesis. Cancer Res..

[21] Wawro M.E., Chojnacka K., Wieczorek-Szukała K., Sobierajska K., Niewiarowska J. (2018). Invasive Colon Cancer Cells Induce Transdifferentiation of Endothelium to Cancer-Associated Fibroblasts through Microtubules Enriched in Tubulin-β3. Int. J. Mol. Sci..

[22] Fan C.S., Chen W.S., Chen L.L., Chen C.C., Hsu Y.T., Chua K.V., Wang H.D., Huang T.S. (2018). Osteopontin-integrin engagement induces HIF-1α-TCF12-mediated endothelial-mesenchymal transition to exacerbate colorectal cancer. Oncotarget.

[23] Zhang T., Zhang L., Gao Y., Wang Y., Liu Y., Zhang H., Wang Q., Hu F., Li J., Tan J. (2021). Role of aneuploid circulating tumor cells and CD31(þ) circulating tumor endothelial cells in predicting and monitoring antiangiogenic therapy efficacy in advanced NSCLC. Mol. Oncol..

[24] Ghafoor S., Garcia E., Jay D.J., Persad S. (2025). Molecular Mechanisms Regulating Epithelial Mesenchymal Transition (EMT) to Promote Cancer Progression. Int. J. Mol. Sci..

[25] Hao Y.M., Yuan H.Q., Ren Z., Qu S.L., Liu L.S., Dang-Heng W., Yin K., Fu M., Jiang Z.S. (2019). Endothelial to mesenchymal transition in atherosclerotic vascular remodeling. Clin. Chim. Acta.

[26] Fontana R., Mestre-Farrera A., Yang J. (2024). Update on Epithelial-Mesenchymal Plasticity in Cancer Progression. Annu. Rev. Pathol..

[27] Yang J., Antin P., Berx G., Brabletz T., Bronner M., Campbell K., Cano A., Casanova J., Christofori G., Dedhar S. (2020). Guidelines and definitions for research on epithelial–mesenchymal transition. Nat. Rev. Mol. Cell Biol..

[28] Van Meeteren L.A., ten Dijke P. (2012). Regulation of endothelial cell plasticity by TGF-β. Cell Tissue Res..

[29] Xiao L., Kim D.J., Davis C.L., McCann J.V., Dunleavey J.M., Vanderlinden A.K., Xu N., Pattenden S.G., Frye S.V., Xu X. (2015). Tumour endothelial cells with distinct patterns of TGFβ-driven endothelial-to-mesenchymal transition. Cancer Res..

[30] Pinto M.T., Ferreira Melo F.U., Malta T.M., Rodrigues E.S., Plaça J.R., Silva W.A., Panepucci R.A., Covas D.T., de Oliveira Rodrigues C., Kashima S. (2018). Endothelial cells from different anatomical origin have distinct responses during SNAIL/TGF-β2-mediated endothelial-mesenchymal transition. Am. J. Transl. Res..

[31] Ferreira F.U., Eduardo Botelho Souza L., Hassibe Thomé C., Tomazini Pinto M., Origassa C., Salustiano S., Marcel Faça V., Olsen Câmara N., Kashima S., Tadeu Covas D. (2019). Endothelial cells tissue-specific origins affects their responsiveness to TGF-β2 during endothelial-to-mesenchymal transition. Int. J. Mol. Sci..

[32] Li Z.X., Chen J.X., Zheng Z.J., Cai W.J., Yang X.B., Huang Y.Y., Gong Y., Xu F., Chen Y.S., Lin L. (2022). TGF-β1 promotes human breast cancer angiogenesis and malignant behavior by regulating endothelial-mesenchymal transition. Front. Oncol..

[33] Lin Q.Q., Zhao J., Zheng C.G., Chun J. (2018). Roles of notch signaling pathway and endothelial-mesenchymal transition in vascular endothelial dysfunction and atherosclerosis. Eur. Rev. Med. Pharmacol. Sci..

[34] Nusse R., Clevers H. (2017). Wnt/β-Catenin Signaling, Disease, and Emerging Therapeutic Modalities. Cell.

[35] Foulquier S., Daskalopoulos E.P., Lluri G., Hermans K.C.M., Deb A., Blankesteijn W.M. (2018). WNT Signaling in Cardiac and Vascular Disease. Pharmacol. Rev..

[36] Wu D.M., Liu T., Deng S.H., Han R., Zhang T., Li J., Xu Y. (2020). Alpha-1 antitrypsin induces epithelial-to-mesenchymal transition, endothelial-to-mesenchymal transition, and drug resistance in lung cancer cells. Onco Targets Ther..

[37] Liu T., Ma W., Xu H., Huang M., Zhang D., He Z., Zhang L., Brem S., O’Rourke D.M., Gong Y. (2018). PDGF-mediated mesenchymal transformation renders endothelial resistance to anti-VEGF treatment in glioblastoma. Nat. Commun..

[38] Mahler G.J., Farrar E.J., Butcher J.T. (2013). Inflammatory cytokines promote mesenchymal transformation in embryonic and adult valve endothelial cells. Arterioscler. Thromb. Vasc. Biol..

[39] Motallebnejad P., Rajesh V.V., Azarin S.M. (2022). Evaluating the Role of IL-1β in Transmigration of Triple Negative Breast Cancer Cells Across the Brain Endothelium. Cell Mol. Bioeng..

[40] Yoshimatsu Y., Wakabayashi I., Kimuro S., Takahashi N., Takahashi K., Kobayashi M., Maishi N., Podyma-Inoue K.A., Hida K., Miyazono K. (2020). TNF-α enhances TGF-β-induced endothelial-to-mesenchymal transition via TGF-β signal augmentation. Cancer Sci..

[41] Adjuto-Saccone M., Soubeyran P., Garcia J., Audebert S., Camoin L., Rubis M., Roques J., Binétruy B., Iovanna J.L., Tournaire R. (2021). TNF-α induces endothelial-mesenchymal transition promoting stromal development of pancreatic adenocarcinoma. Cell Death Dis..

[42] Montorfano I., Becerra A., Cerro R., Echeverría C., Sáez E., Morales M.G., Fernández R., Cabello-Verrugio C., Simon F. (2014). Oxidative stress mediates the conversion of endothelial cells into myofibroblasts via a TGF-β1 and TGF-β2-dependent pathway. Lab. Investig..

[43] Doerr M., Morrison J., Bergeron L., Coomber B.L., Viloria-Petit A. (2016). Differential effect of hypoxia on early endothelial-mesenchymal transition response to transforming growth beta isoforms 1 and 2. Microvasc. Res..

[44] Zhang B., Niu W., Dong H.Y., Liu M.L., Luo Y., Li Z.C. (2018). Hypoxia induces endothelial mesenchymal transition in pulmonary vascular remodeling. Int. J. Mol. Med..

[45] Banerjee D., Barton S.M., Grabham P.W., Rumeld A.L., Okochi S., Street C., Kadenhe-Chiweshe A., Boboila S., Yamashiro D.J., Connolly E.P. (2020). High-Dose Radiation Increases Notch1 in Tumor vasculature. Int. J. Radiat. Oncol. Biol. Phys..

[46] Bouten R.M., Dalgard C.L., Soltis A.R., Slaven J.E., Day R.M. (2021). Transcriptomic profiling and pathway analysis of cultured human lung microvascular endothelial cells following ionizing radiation exposure. Sci. Rep..

[47] Lai C., Wu Z., Li Z., Huang X., Hu Z., Yu H., Yuan Z., Shi J., Hu J., Mulati Y. (2024). Single-cell analysis extracted CAFs-related genes to established online app to predict clinical outcome and radiotherapy prognosis of prostate cancer. Clin. Transl. Oncol..

[48] Wu D., Deng S., Li L., Liu T., Zhang T., Li J., Yu Y., Xu Y. (2021). TGF-β1-mediated exosomal lnc-MMP2-2 increases blood-brain barrier permeability via the miRNA-1207-5p/EPB41L5 axis to promote non-small cell lung cancer brain metastasis. Cell Death Dis..

[49] Shi X., Yang J., Deng S., Xu H., Wu D., Zeng Q., Wang S., Hu T., Wu F., Zhou H. (2022). TGF-β signaling in the tumour metabolic microenvironment and targeted therapies. BioMed Central.

[50] Merk L., Regel K., Eckhardt H., Evers M., El-Ayoubi A., Mittelbronn M., Krüger M., Gérardy J.J., Mack A.F., Naumann U. (2024). Blocking TGF-β and Epithelial-to-Mesenchymal Transition (EMT)-mediated activation of vessel-associated mural cells in glioblastoma impacts tumour angiogenesis. Free Neuropathol..

[51] Deng Z., Fan T., Xiao C., Tian H., Zheng Y., Li C., He J. (2024). TGF-β signaling in health, disease, and therapeutics. Signal Transduct. Target. Ther..

[52] Zeisberg E.M., Tarnavski O., Zeisberg M., Dorfman A.L., McMullen J.R., Gustafsson E., Chandraker A., Yuan X., Pu W.T., Roberts A.B. (2007). Endothelial-to-mesenchymal transition contributes to cardiac fibrosis. Nat. Med..

[53] Goumans M.J., van Zonneveld A.J., ten Dijke P. (2008). Transforming growth factor β-induced endothelial-to-mesenchymal transition: A switch to cardiac fibrosis?. Trends Cardiovasc. Med..

[54] Li J., Qu X., Yao J., Caruana G., Ricardo S.D., Yamamoto Y., Yamamoto H., Bertram J.F. (2010). Blockade of endothelial-mesenchymal transition by a Smad3 inhibitor delays the early development of streptozotocin-induced diabetic nephropathy. Diabetes.

[55] Medici D., Potenta S., Kalluri R. (2011). Transforming growth factor-β2 promotes Snail-mediated endothelial-mesenchymal transition through convergence of Smad-dependent and Smad-independent signalling. Biochem. J..

[56] Li Z., Wang F., Zha S., Cao Q., Sheng J., Chen S. (2018). SIRT1 inhibits TGF-_-induced endothelial-mesenchymal transition in human endothelial cells with Smad4 deacetylation. J. Cell Physiol..

[57] Lin J.R., Zheng Y.J., Zhang Z.B., Shen W.L., Li X.D., Wei T., Ruan C.C., Chen X.H., Zhu D.L., Gao P.J. (2018). Suppression of Endothelial-to-Mesenchymal Transition by SIRT (Sirtuin) 3 Alleviated the Development of Hypertensive Renal Injury. Hypertension.

[58] Lambers C., Roth M., Zhong J., Campregher C., Binder P., Burian B., Petkov V., Block L.H. (2013). The interaction of endothelin-1 and TGF-beta1 mediates vascular cell remodeling. PLoS ONE.

[59] Wermuth P.J., Li Z., Mendoza F.A., Jimenez S.A. (2016). Stimulation of Transforming Growth Factor-β1-Induced Endothelial-To-Mesenchymal Transition and Tissue Fibrosis by Endothelin-1 (ET-1): A Novel Profibrotic Effect of ET-1. PLoS ONE.

[60] Pang L., Yang S., Dai W., Wu S., Kong J. (2022). Role of caveolin-1 in human organ function and disease: Friend or foe?. Carcinogenesis.

[61] Becerra A., Rojas M., Vallejos A., Villegas V., Pérez L., Cabello-Verrugio C., Simon F. (2017). Endothelial fibrosis induced by suppressed STAT3 expression mediated by signaling involving the TGF-β1/ALK5/Smad pathway. Lab. Investig..

[62] Krizbai I.A., Gasparics Á., Nagy’’oszi P., Fazakas C., Molnár J., Wilhelm I., Bencs R., Rosivall L., Sebe A. (2015). Endothelial-mesenchymal transition of brain endothelial cells: Possible role during metastatic extravasation. PLoS ONE.

[63] Yin Z., Dong C., Jiang K., Xu Z., Li R., Guo K., Shao S., Wang L. (2019). Heterogeneity of cancer-associated fibroblasts and roles in the progression, prognosis, and therapy of hepatocellular carcinoma. J. Hematol. Oncol..

[64] Wu D.M., Deng S.H., Zhou J., Han R., Liu T., Zhang T., Li J., Chen J.P., Xu Y. (2020). PLEK2 mediates metastasis and vascular invasion via the ubiquitin-dependent degradation of SHIP2 in non-small cell lung cancer. Int. J. Cancer.

[65] Kim S.H., Song Y., Seo H.R. (2019). GSK-3beta regulates the endothelialto-mesenchymal transition via reciprocal crosstalk between NSCLC cells and HUVECs in multicellular tumor spheroid models. J. Exp. Clin. Cancer Res..

[66] Marin-Ramos N.I., Jhaveri N., Thein T.Z., Fayngor R.A., Chen T.C., Hofman F.M. (2019). NEO212, a conjugate of temozolomide and perillyl alcohol, blocks the endothelial-to-mesenchymal transition in tumor-associated brain endothelial cells in glioblastoma. Cancer Lett..

[67] Shi Q., Xue C., Zeng Y., Yuan X., Chu Q., Jiang S., Wang J., Zhang Y., Zhu D., Li L. (2024). Notch signaling pathway in cancer: From mechanistic insights to targeted therapies. Signal Transduct. Target. Ther..

[68] Guan S., Zhou J. (2017). CXCR7 attenuates the TGF-_-induced endothelial-to-mesenchymal transition and pulmonary fibrosis. Mol. Biosyst..

[69] Patel J., Baz B., Wong H.Y., Lee J.S., Khosrotehrani K. (2018). Accelerated Endothelial to Mesenchymal Transition Increased Fibrosis via Deleting Notch Signaling in Wound Vasculature. J. Investig. Dermatol..

[70] Wang S.H., Chang J.S., Hsiao J.R., Yen Y.C., Jiang S.S., Liu S.H., Chen Y.L., Shen Y.Y., Chang J.Y., Chen Y.W. (2017). Tumour cell-derived WNT5B modulates in vitro lymphangiogenesis via induction of partial endothelial-mesenchymal transition of lymphatic endothelial cells. Oncogene.

[71] Huang M., Zhang D., Wu J.Y., Xing K., Yeo E., Li C., Zhang L., Holland E., Yao L., Qin L. (2020). Wnt mediated endothelial transformation into mesenchymal stem cell-like cells induces chemoresistance in glioblastoma. Sci. Transl. Med..

[72] Yu F., Yu C., Li F., Zuo Y., Wang Y., Yao L., Wu C., Wang C., Ye L. (2021). Wnt/β-catenin signaling in cancers and targeted therapies. Signal Transduct. Target. Ther..

[73] Piera-Velazquez S., Jimenez S.A. (2015). Role of cellular senescence and NOX4-mediated oxidative stress in systemic sclerosis pathogenesis. Curr. Rheumatol. Rep..

[74] Echeverría C., Montorfano I., Sarmiento D., Becerra A., Nuñez-Villena F., Figueroa X.F., Cabello-Verrugio C., Elorza A.A., Riedel C., Simon F. (2013). Lipopolysaccharide induces a fibrotic-like phenotype in endothelial cells. J. Cell Mol. Med..

[75] Li J., Zhang Q., Ren C., Wu X., Zhang Y., Bai X., Lin Y., Li M., Fu J., Kopylov P. (2018). Low-Intensity Pulsed Ultrasound Prevents the Oxidative Stress Induced Endothelial-Mesenchymal Transition in Human Aortic Endothelial Cells. Cell Physiol. Biochem..

[76] Xu X., Tan X., Tampe B., Sanchez E., Zeisberg M., Zeisberg E.M. (2015). Snail Is a Direct Target of Hypoxia-inducible Factor 1alpha (HIF1alpha) in Hypoxia-induced Endothelial to Mesenchymal Transition of Human Coronary Endothelial Cells. J. Biol. Chem..

[77] Xu X., Tan X., Hulshoff M.S., Wilhelmi T., Zeisberg M., Zeisberg E.M. (2016). Hypoxia-induced endothelial-mesenchymal transition is associated with RASAL1 promoter hypermethylation in human coronary endothelial cells. FEBS Lett..

[78] Zhu P., Huang L., Ge X., Yan F., Wu R., Ao Q. (2006). Transdifferentiation of pulmonary arteriolar endothelial cells into smooth muscle-like cells regulated by myocardin involved in hypoxia-induced pulmonary vascular remodelling. Int. J. Exp. Pathol..

[79] Derada Troletti C., Fontijn R.D., Gowing E., Charabati M., van Het Hof B., Didouh I., van der Pol S.M.A., Geerts D., Prat A., van Horssen J. (2019). Inflammation-induced endothelial to mesenchymal transition promotes brain endothelial cell dysfunction and occurs during multiple sclerosis pathophysiology. Cell Death Dis..

[80] Pérez L., Muñoz-Durango N., Riedel C.A., Echeverría C., Kalergis A.M., Cabello-Verrugio C., Simon F. (2017). Endothelial-to-mesenchymal transition: Cytokine-mediated pathways that determine endothelial fibrosis under inflammatory conditions. Cytokine Growth Factor. Rev..

[81] Fahey E., Doyle S.L. (2019). IL-1 Family Cytokine Regulation of Vascular Permeability and Angiogenesis. Front. Immunol..

[82] Takagi K., Yamakuchi M., Matsuyama T., Kondo K., Uchida A., Misono S., Hashiguchi T., Inoue H. (2018). IL-13 enhances mesenchymal transition of pulmonary artery endothelial cells via down-regulation of miR-424/503 in vitro. Cell Signal.

[83] Chrobak I., Lenna S., Stawski L., Trojanowska M. (2013). Interferon-alpha promotes vascular remodeling in human microvascular endothelial cells by upregulating endothelin (ET)-1 and transforming growth factor (TGF)beta2. J. Cell Physiol..

[84] Yang Y., Luo N.S., Ying R., Xie Y., Chen J.Y., Wang X.Q., Gu Z.J., Mai J.T., Liu W.H., Wu M.X. (2017). Macrophage-derived foam cells impair endothelial barrier function by inducing endothelial-mesenchymal transition via CCL-4. Int. J. Mol. Med..

[85] Kobayashi M., Fujiwara K., Takahashi K., Yoshioka Y., Ochiya T., Podyma-Inoue K.A., Watabe T. (2022). Transforming growth factor-β-induced secretion of extracellular vesicles from oral cancer cells evokes endothelial barrier instability via endothelial-mesenchymal transition. Inflamm. Regen..

[86] Chen P.Y., Qin L., Barnes C., Charisse K., Yi T., Zhang X., Ali R., Medina P.P., Yu J., Slack F.J. (2012). FGF regulates TGF-β signaling and endothelial-to-mesenchymal transition via control of let-7 miRNA expression. Cell Rep..

[87] Chen P.Y., Qin L., Tellides G., Simons M. (2014). Fibroblast growth factor receptor 1 is a key inhibitor of TGFβ signaling in the endothelium. Sci. Signal.

[88] Correia A.C., Moonen J.R., Brinker M.G., Krenning G. (2016). FGF2 inhibits endothelial-mesenchymal transition through microRNA-20a-mediated repression of canonical TGF-β signaling. J. Cell Sci..

[89] Wang Z., Calpe B., Zerdani J., Lee Y., Oh J., Bae H., Khademhosseini A., Kim K. (2016). High-throughput investigation of endothelial-to-mesenchymal transformation (EndMT) with combinatorial cellular microarrays. Biotechnol. Bioeng..

[90] Ichise T., Yoshida N., Ichise H. (2014). FGF2-induced Ras-MAPK signalling maintains lymphatic endothelial cell identity by upregulating endothelial-cell-specific gene expression and suppressing TGFβ signalling through Smad2. J. Cell Sci..

[91] Yang J.H., Wylie-Sears J., Bischoff J. (2008). Opposing actions of Notch1 and VEGF in post-natal cardiac valve endothelial cells. Biochem. Biophys. Res. Commun..

[92] Illigens B.M., Casar Berazaluce A., Poutias D., Gasser R., Del Nido P.J., Friehs I. (2017). Vascular Endothelial Growth Factor Prevents Endothelial-to-Mesenchymal Transition in Hypertrophy. Ann. Thorac. Surg..

[93] Shi S., Kanasaki K., Koya D. (2016). Linagliptin but not Sitagliptin inhibited transforming growth factor-β2-induced endothelial DPP-4 activity and the endothelial-mesenchymal transition. Biochem. Biophys. Res. Commun..

[94] Wang Z., Fei S., Suo C., Han Z., Tao J., Xu Z., Zhao C., Tan R., Gu M. (2018). Antifibrotic Effects of Hepatocyte Growth Factor on Endothelial-to-Mesenchymal Transition via Transforming Growth Factor-Beta1 (TGF-β1)/Smad and Akt/mTOR/P70S6K Signaling Pathways. Ann. Transpl..

[95] Zhang H., Liu Y., Yan L., Du W., Zhang X., Zhang M., Chen H., Zhang Y., Zhou J., Sun H. (2018). Bone morphogenetic protein-7 inhibits endothelial-mesenchymal transition in pulmonary artery endothelial cell under hypoxia. J. Cell Physiol..

[96] Zhang H., Hu J., Liu L. (2017). MiR-200a modulates TGF-beta1-induced endothelial-to-mesenchymal shift via suppression of GRB2 in HAECs. Biomed. Pharmacother..

[97] Sun Y., Cai J., Yu S., Chen S., Li F., Fan C. (2016). MiR-630 Inhibits Endothelial-Mesenchymal Transition by Targeting Slug in Traumatic Heterotopic Ossification. Sci. Rep..

[98] Kanasaki K., Shi S., Kanasaki M. (2014). Linagliptin-mediated DPP-4 inhibition ameliorates kidney fibrosis in streptozotocin-induced diabetic mice by inhibiting endothelial-to-mesenchymal transition in a therapeutic regimen. Diabetes.

[99] Zhang S., Weinheimer C., Courtois M. (2003). The role of the Grb2-p38 MAPK signaling pathway in cardiac hypertrophy and fibrosis. J. Clin. Investig..

[100] Ge S., Xie J., Liu F., He J., He J. (2015). MicroRNA-19b reduces hepatic stellate cell proliferation by targeting GRB2 in hepatic fibrosis models in vivo and in vitro as part of the inhibitory effect of estradiol. J. Cell Biochem..

[101] Papetti M., Shujath J., Riley K.N., Herman I.M. (2003). FGF-2 antagonizes the TGF-beta1-mediated induction of pericyte alpha-smooth muscle actin expression: A role for myf-5 and Smad-mediated signaling pathways. Investig. Ophthalmol. Vis. Sci..

[102] Ramos C., Becerril C., Montano M. (2010). FGF-1 reverts epithelial-mesenchymal transition induced by TGF-{beta}1 through MAPK/ERK kinase pathway. Am. J. Physiol. Lung Cell Mol. Physiol..

[103] Miscianinov V., Martello A., Rose L., Parish E., Cathcart B., Mitic´ T., Gray G.A., Meloni M., Al Haj Zen A., Caporali A. (2018). MicroRNA-148b Targets the TGF-B Pathway to Regulate Angiogenesis and Endothelial-to-Mesenchymal Transition during Skin Wound Healing. Mol. Ther..

[104] Lagendijk A.K., Goumans M.J., Burkhard S.B. (2011). MicroRNA-23 restricts cardiac valve formation by inhibiting Has2 and extracellular hyaluronic acid production. Circ. Res..

[105] Bayoumi A.S., Teoh J.P., Aonuma T. (2017). MicroRNA-532 protects the heart in acute myocardial infarction, and represses prss23, a positive regulator of endothelial-to-mesenchymal transition. Cardiovasc. Res..

[106] Bijkerk R., de Bruin R.G., van Solingen C. (2012). MicroRNA-155 functions as a negative regulator of RhoA signaling in TGF-beta-induced endothelial to mesenchymal transition. Microrna.

[107] Feng B., Cao Y., Chen S., Chu X., Chu Y., Chakrabarti S. (2016). miR-200b Mediates Endothelial-to-Mesenchymal Transition in Diabetic Cardiomyopathy. Diabetes.

[108] Geng H.Z., Guan J. (2017). MiR-18a-5p inhibits endothelial mesenchymal transition and cardiac fibrosis through the Notch2 pathway. Biochem. Biophys. Res. Commun..

[109] Kumarswamy R., Volkmann I., Jazbutyte V., Dangwal S., Park D.H., Thum T. (2012). Transforming Growth Factor-beta-Induced Endothelial-to-Mesenchymal Transition Is Partly Mediated by MicroRNA-21. Arterioscler. Thromb. Vasc. Biol..

[110] Ghosh A.K., Nagpal V., Covington J.W., Michaels M.A., Vaughan D.E. (2012). Molecular basis of cardiac endothelial-to-mesenchymal transition (EndMT): Differential expression of microRNAs during EndMT. Cell Signal.

[111] Suzuki H.I., Katsura A., Mihira H., Horie M., Saito A., Miyazono K. (2017). Regulation of TGF-beta-mediated endothelial-mesenchymal transition by microRNA-27. J. Biochem..

[112] Li L., Kim I.K., Chiasson V., Chatterjee P., Gupta S. (2017). NF-kappaB mediated miR-130a modulation in lung microvascular cell remodeling: Implication in pulmonary hypertension. Exp. Cell Res..

[113] Xu Y.P., He Q., Shen Z. (2017). MiR-126a-5p is involved in the hypoxia-induced endothelial-to-mesenchymal transition of neonatal pulmonary hypertension. Hypertens. Res..

[114] Hulshoff M.S., Xu X., Krenning G., Zeisberg E.M. (2018). Epigenetic regulation of endothelial-to-mesenchymal transition in chronic heart Disease. Arterioscler. Thromb. Vasc. Biol..

[115] Wen B., Tao R., Liu Y., Zhang Z. (2024). Investigating the role of exosomal microRNA-5703 in modulating tumor-associated endothelial cells in lung cancer. Cytojournal.

[116] Jordan N.P., Tingle S.J., Shuttleworth V.G., Cooke K., Redgrave R.E., Singh E., Glover E.K., Ahmad Tajuddin H.B., Kirby J.A., Arthur H.M. (2021). MiR-126-3p Is Dynamically Regulated in Endothelial-to-Mesenchymal Transition during Fibrosis. Int. J. Mol. Sci..

[117] Neumann P., Jaé N., Knau A., Glaser S.F., Fouani Y., Rossbach O., Krüger M., John D., Bindereif A., Grote P. (2018). The lncRNA GATA6-AS epigenetically regulates endothelial gene expression via interaction with LOXL2. Nat. Commun..

[118] Hu J.X., Zheng Z.Q., Kang T., Qian W., Huang S.H., Li B.G. (2022). LncRNA LINC00961 regulates endothelial mesenchymal transition via the PTEN PI3K AKT pathway. Mol. Med. Rep..

[119] Xiang Y., Zhang Y., Tang Y., Li Q. (2017). MALAT1 modulates tgf-beta1-induced endothelial-to-mesenchymal transition through downregulation of miR-145. Cell Physiol. Biochem..

[120] Yu X., Huang J., Liu X., Li J., Yu M., Li M., Xie Y., Li Y., Qiu J., Xu Z. (2024). LncRNAH19 acts as a ceRNA of let-7 g to facilitate endothelial-to-mesenchymal transition in hypoxic pulmonary hypertension via regulating TGF-β signalling pathway. Respir. Res..

[121] Fan C.S., Chen L.L., Hsu T.A., Chen C.C., Chua K.V., Li C.P., Huang T.S. (2019). Endothelial-mesenchymal transition harnesses HSP90α-secreting M2-macrophages to exacerbate pancreatic ductal adenocarcinoma. J. Hematol. Oncol..

[122] Yi M., Liu B., Tang Y., Li F., Qin W., Yuan X. (2018). Irradiated Human Umbilical Vein Endothelial Cells Undergo Endothelial-Mesenchymal Transition via the Snail/miR-199a-5p Axis to Promote the Differentiation of Fibroblasts into Myofibroblasts. Biomed. Res. Int..

[123] Choi S.H., Hong Z.Y., Nam J.K., Lee H.J., Jang J., Yoo R.J., Lee Y.J., Lee C.Y., Kim K.H., Park S. (2015). A Hypoxia-Induced Vascular Endothelial-to-Mesenchymal Transition in Development of Radiation-Induced Pulmonary Fibrosis. Clin. Cancer Res..

[124] Mintet E., Rannou E., Buard V., West G., Guipaud O., Tarlet G., Sabourin J.C., Benderitter M., Fiocchi C., Milliat F. (2015). Identification of Endothelial-to-Mesenchymal Transition as a Potential Participant in Radiation Proctitis. Am. J. Pathol..

[125] Ciszewski W.M., Sobierajska K., Wawro M.E., Klopocka W., Chefczyńska N., Muzyczuk A., Siekacz K., Wujkowska A., Niewiarowska J. (2017). The ILK-MMP9-MRTF axis is crucial for EndMT differentiation of endothelial cells in a tumor microenvironment. Biochim. Biophys. Acta Mol. Cell Res..

[126] Zhen J., Jiao K., Yang K., Wu M., Zhou Q., Yang B., Xiao W., Hu C., Zhou M., Li Z. (2021). The 14-3-3η/GSK-3β/β-catenin complex regulates EndMT induced by 27-hydroxycholesterol in HUVECs and promotes the migration of breast cancer cells. Cell Biol. Toxicol..

[127] Chen J., Han S., Chen J., Hu P., Zeng Z., Hu Y., Xiong H., Ke Z., Zhang Y., Xu F. (2021). reciprocal feedback of miR-548ac/YB-1/Snail induces EndMT of HUVECs during acidity microenvironment. Cancer Cell Int..

[128] Dudley A.C., Khan Z.A., Shih S.C., Kang S.Y., Zwaans B.M., Bischoff J., Klagsbrun M. (2008). Calcification of multipotent prostate tumor endothelium. Cancer Cell.

[129] Smeda M., Kieronska A., Adamski M.G., Proniewski B., Sternak M., Mohaissen T., Przyborowski K., Derszniak K., Kaczor D., Stojak M. (2018). Nitric oxide deficiency and endothelial-mesenchymal transition of pulmonary endothelium in the progression of 4T1 metastatic breast cancer in mice. Breast Cancer Res..

[130] Thannickal V.J., Henke C.A., Horowitz J.C., Noble P.W., Roman J., Sime P.J., Zhou Y., Wells R.G., White E.S., Tschumperlin D.J. (2014). Matrix biology of idiopathic pulmonary fibrosis: A workshop report of the national heart, lung, and blood institute. Am. J. Pathol..

[131] Rieder F., Kessler S.P., West G.A., Bhilocha S., de la Motte C., Sadler T.M., Gopalan B., Stylianou E., Fiocchi C. (2011). Inflammation-induced endothelial-to-mesenchymal transition: A novel mechanism of intestinal fibrosis. Am. J. Pathol..

[132] Wang G., Yang Q., Li M., Zhang Y., Cai Y., Liang X., Fu Y., Xiao Z., Zhou M., Xie Z. (2019). Quantitative proteomic profiling of tumor-associated vascular endothelial cells in colorectal cancer. Biol. Open.

[133] Wang H., Feng C., Lu M., Zhang B., Xu Y., Zeng Q., Xi J., Zhou J., Ying X., Zhang J. (2021). Integrative single-cell transcriptome analysis reveals a subpopulation of fibroblasts associated with favorable prognosis of liver cancer patients. Transl. Oncol..

[134] Cai W., Sun X., Jin F., Xiao D., Li H., Sun H., Wang Y., Lu Y., Liu J., Huang C. (2021). PERK-eIF2α-ERK1/2 axis drives mesenchymal-endothelial transition of cancer-associated fibroblasts in pancreatic cancer. Cancer Lett..

[135] Hashimoto N., Phan S.H., Imaizumi K., Matsuo M., Nakashima H., Kawabe T., Shimokata K., Hasegawa Y. (2010). Endothelial-mesenchymal transition in bleomycin-induced pulmonary fibrosis. Am. J. Respir. Cell Mol. Biol..

[136] Jonker L., Arthur H.M. (2002). Endoglin expression in early development is associated with vasculogenesis and angiogenesis. Mech. Dev..

[137] Bernabeu C., Conley B.A., Vary C.P. (2007). Novel biochemical pathways of endoglin in vascular cell physiology. J. Cell Biochem..

[138] Jerkic M., Rodríguez-Barbero A., Prieto M., Toporsian M., Pericacho M., Rivas-Elena J.V., Obreo J., Wang A., Pérez-Barriocanal F., Arévalo M. (2006). Reduced angiogenic responses in adult Endoglin heterozygous mice. Cardiovasc. Res..

[139] Anderberg C., Cunha S.I., Zhai Z., Cortez E., Pardali E., Johnson J.R., Franco M., Páez-Ribes M., Cordiner R., Fuxe J. (2013). Deficiency for endoglin in tumor vasculature weakens the endothelial barrier to metastatic dissemination. J. Exp. Med..

[140] Chauhdari T., Zaidi S.A., Su J., Ding Y. (2025). Organoids meet microfluidics: Recent advancements, challenges, and future of organoids-on-chip. In Vitro Models.

[141] Wang P., Wu Y., Chen W., Zhang M., Qin J. (2022). Malignant Melanoma-Derived Exosomes Induce Endothelial Damage and Glial Activation on a Human BBB Chip Model. Biosensors.

[142] Jeong K., Yu Y.J., You J.Y., Rhee W.J., Kim J.A. (2020). Exosome-mediated microRNA-497 delivery for anti-cancer therapy in a microfluidic 3D lung cancer model. Lab. Chip..

[143] Whiteford J., Arokiasamy S., Thompson C.L., Dufton N.P. (2022). Novel application of live imaging to determine the functional cell biology of endothelial-to-mesenchymal transition (EndMT) within a liver-on-a-chip platform. In Vitro Models.

[144] Plebani R., Potla R., Soong M., Bai H., Izadifar Z., Jiang A., Travis R.N., Belgur C., Dinis A., Cartwright M.J. (2022). Modeling pulmonary cystic fibrosis in a human lung airway-on-a-chip. J. Cyst. Fibros..

[145] Kramer B., Corallo C., van den Heuvel A., Crawford J., Olivier T., Elstak E., Giordano N., Vulto P., Lanz H.L., Janssen R.A.J. (2022). High-throughput 3D microvessel-on-a-chip model to study defective angiogenesis in systemic sclerosis. Sci. Rep..

[146] Hernández-Camarero P., Toledo B., Diaz-Ruano A.B., González-Titos A., García-Ortega M.B., Perán M. (2025). What is the Impact of Endothelial-to-Mesenchymal Transition in Solid Tumours: A Qualitative Systematic Review and Quantitative Meta-Analysis. Int. J. Biol. Sci..

[147] Welch-Reardon K.M., Ehsan S.M., Wang K., Wu N., Newman A.C., Romero-Lopez M., Fong A.H., George S.C., Edwards R.A., Hughes C.C. (2014). Angiogenic sprouting is regulated by endothelial cell expression of Slug. J. Cell Sci..

[148] Folkman J. (1971). Tumor angiogenesis: Therapeutic implications. N. Engl. J. Med..

[149] Gu A., Jie Y., Yao Q., Zhang Y., Mingyan E. (2017). Slug Is Associated with Tumor Metastasis and Angiogenesis in Ovarian Cancer. Reprod. Sci..

[150] Enholm B., Paavonen K., Ristimäki A., Kumar V., Gunji Y., Klefstrom J., Kivinen L., Laiho M., Olofsson B., Joukov V. (1997). Comparison of VEGF, VEGF-B, VEGF-C and Ang-1 mRNA regulation by serum, growth factors, oncoproteins and hypoxia. Oncogene.

[151] Ferrari G., Cook B.D., Terushkin V., Pintucci G., Mignatti P. (2009). Transforming growth factor-beta 1 (TGF-beta1) induces angiogenesis through vascular endothelial growth factor (VEGF)-mediated apoptosis. J. Cell Physiol..

[152] Armulik A., Abramsson A., Betsholtz C. (2005). Endothelial/pericyte interactions. Circ. Res..

[153] Gasparics Á., Rosivall L., Krizbai I.A., Sebe A. (2016). When the endothelium scores an own goal: Endothelial cells actively augment metastatic extravasation through endothelial-mesenchymal transition. Am. J. Physiol. Heart Circ. Physiol..

[154] Helfrich I., Scheffrahn I., Bartling S., Weis J., von Felbert V., Middleton M., Kato M., Ergün S., Augustin H.G., Schadendorf D. (2010). Resistance to antiangiogenic therapy is directed by vascular phenotype, vessel stabilization, and maturation in malignant melanoma. J. Exp. Med..

[155] Franco M., Roswall P., Cortez E., Hanahan D., Pietras K. (2011). Pericytes promote endothelial cell survival through induction of autocrine VEGF-A signaling and Bcl-w expression. Blood..

[156] Guo T., Xu J. (2024). Cancer-associated fibroblasts: A versatile mediator in tumor progression, metastasis, and targeted therapy. Cancer Metastasis Rev..

[157] Sahai E., Astsaturov I., Cukierman E., DeNardo D.G., Egeblad M., Evans R.M., Fearon D., Greten F.R., Hingorani S.R., Hunter T. (2020). A framework for advancing our understanding of cancer-associated fibroblasts. Nat. Rev. Cancer.

[158] Natesh N.R., Mogha P., Chen A., Antonia S.J., Varghese S. (2024). Differential roles of normal and lung cancer-associated fibroblasts in microvascular network formation. APL Bioeng..

[159] Fang J., Lu Y., Zheng J., Jiang X., Shen H., Shang X., Lu Y., Fu P. (2023). Exploring the crosstalk between endothelial cells, immune cells, and immune checkpoints in the tumor microenvironment: New insights and therapeutic implications. Cell Death Dis..

[160] Amersfoort J., Eelen G., Carmeliet P. (2022). Immunomodulation by endothelial cells—Partnering up with the immune system?. Nat. Rev. Immunol..

[161] Daum S., Decristoforo L., Mousa M., Salcher S., Plattner C., Hosseinkhani B., Trajanoski Z., Wolf D., Carmeliet P., Pircher A. (2025). Unveiling the immunomodulatory dance: Endothelial cells’ function and their role in non-small cell lung cancer. Mol. Cancer.

[162] Goveia J., Rohlenova K., Taverna F., Treps L., Conradi L.C., Pircher A., Geldhof V., de Rooij L.P.M.H., Kalucka J., Sokol L. (2020). An Integrated Gene Expression Landscape Profiling Approach to Identify Lung Tumor Endothelial Cell Heterogeneity and Angiogenic Candidates. Cancer Cell.

[163] Sozio F., Schioppa T., Laffranchi M., Salvi V., Tamassia N., Bianchetto-Aguilera F.M., Tiberio L., Bonecchi R., Bosisio D., Parmentier M. (2023). CCRL2 Expression by Specialized Lung Capillary Endothelial Cells Controls NK-cell Homing in Lung Cancer. Cancer Immunol. Res..

[164] Thompson T.W., Kim A.B., Li P.J., Wang J., Jackson B.T., Huang K.T.H., Zhang L., Raulet D.H. (2017). Endothelial cells express NKG2D ligands and desensitize antitumor NK responses. Elife.

[165] Pieterse E., Rother N., Garsen M., Hofstra J.M., Satchell S.C., Hoffmann M., Loeven M.A., Knaapen H.K., van der Heijden O.W.H., Berden J.H.M. (2017). Neutrophil Extracellular Traps Drive Endothelial-to-Mesenchymal Transition. Arterioscler. Thromb. Vasc. Biol..

[166] Jiang Z.Z., Peng Z.P., Liu X.C., Guo H.F., Zhou M.M., Jiang D., Ning W.R., Huang Y.F., Zheng L., Wu Y. (2022). Neutrophil extracellular traps induce tumor metastasis through dual effects on cancer and endothelial cells. Oncoimmunology.

[167] Yang S., Sun B., Li J., Li N., Zhang A., Zhang X., Yang H., Zou X. (2023). Neutrophil extracellular traps promote angiogenesis in gastric cancer. Cell Commun. Signal.

[168] Xie L., Yin J., Kong H., Yu J., Sun M., Wang X., Bai C., Song Y., Yang D. (2023). The Involvement of Tumor Endothelial Cells in the Regulation of PD-L1 and Tregs in the Immune Microenvironment of Early-stage Lung Adenocarcinoma. J. Thorac. Oncol..

[169] Dong P., Xiong Y., Yue J., Hanley S.J.B., Watari H. (2018). B7H3 As a Promoter of Metastasis and Promising Therapeutic Target. Front. Oncol..

[170] Kontos F., Michelakos T., Kurokawa T., Sadagopan A., Schwab J.H., Ferrone C.R., Ferrone S. (2021). B7-H3: An Attractive Target for Antibody-based Immunotherapy. Clin. Cancer Res..

[171] Bruno T.C., Ebner P.J., Moore B.L., Squalls O.G., Waugh K.A., Eruslanov E.B., Singhal S., Mitchell J.D., Franklin W.A., Merrick D.T. (2017). Antigen-Presenting Intratumoral B Cells Affect CD4^+^ TIL Phenotypes in Non-Small Cell Lung Cancer Patients. Cancer Immunol. Res..

[172] Yang C., Lee H., Pal S., Jove V., Deng J., Zhang W., Hoon D.S., Wakabayashi M., Forman S., Yu H. (2013). B cells promote tumor progression via STAT3 regulated-angiogenesis. PLoS ONE.

[173] Sakano Y., Noda T., Kobayashi S., Sasaki K., Iwagami Y., Yamada D., Tomimaru Y., Akita H., Gotoh K., Takahashi H. (2022). Tumor endothelial cell-induced CD8^+^ T-cell exhaustion via GPNMB in hepatocellular carcinoma. Cancer Sci..

[174] Zhang J., Lu T., Lu S., Ma S., Han D., Zhang K., Xu C., Liu S., Gan L., Wu X. (2022). Single-cell analysis of multiple cancer types reveals differences in endothelial cells between tumors and normal tissues. Comput. Struct. Biotechnol. J..

[175] Mori M., Sakamoto A., Kawakami R., Guo L., Slenders L., Mosquera J.V., Ghosh S.K.B., Wesseling M., Shiraki T., Bellissard A. (2024). CD163^+^ Macrophages Induce Endothelial-to-Mesenchymal Transition in Atheroma. Circ. Res..

[176] Maleszewska M., Moonen J.R., Huijkman N., van de Sluis B., Krenning G., Harmsen M.C. (2013). IL-1β and TGFβ2 synergistically induce endothelial to mesenchymal transition in an NFκB-dependent manner. Immunobiology.

[177] Bronson R., Lyu J., Xiong J. (2023). Transcriptome analysis reveals molecular signature and cell-type difference of Homo sapiens endothelial-to-mesenchymal transition. G3.

[178] Mao X., Xu J., Wang W., Liang C., Hua J., Liu J., Zhang B., Meng Q., Yu X., Shi S. (2021). Crosstalk between cancer-associated fibroblasts and immune cells in the tumor microenvironment: New findings and future perspectives. Mol. Cancer.

[179] Gerstberger S., Jiang Q., Ganesh K. (2023). Metastasis. Cell.

[180] Sun H., Breslin J.W., Zhu J., Yuan S.Y., Wu M.H. (2006). Rho and ROCK signaling in VEGF-induced microvascular endothelial hyperpermeability. Microcirculation.

[181] Li B., Zhao W.D., Tan Z.M., Fang W.G., Zhu L., Chen Y.H. (2006). Involvement of Rho/ROCK signalling in small cell lung cancer migration through human brain microvascular endothelial cells. FEBS Lett..

[182] Li D.K., Chen X.R., Wang L.N., Wang J.H., Li J.K., Zhou Z.Y., Li X., Cai L.B., Zhong S.S., Zhang J.J. (2022). Exosomal HMGA2 protein from EBV-positive NPC cells destroys vascular endothelial barriers and induces endothelial-to-mesenchymal transition to promote metastasis. Cancer Gene Ther..

[183] Tanaka M., Koyama T., Sakurai T., Kamiyoshi A., Ichikawa-Shindo Y., Kawate H., Liu T., Xian X., Imai A., Zhai L. (2016). The endothelial adrenomedullin-RAMP2 system regulates vascular integrity and suppresses tumour metastasis. Cardiovasc. Res..

[184] Bhat G.R., Sethi I., Sadida H.Q., Rah B., Mir R., Algehainy N., Albalawi I.A., Masoodi T., Subbaraj G.K., Jamal F. (2024). Cancer cell plasticity: From cellular, molecular, and genetic mechanisms to tumor heterogeneity and drug resistance. Cancer Metastasis Rev..

[185] Murugavel S., Bugyei-Twum A., Matkar P.N., Al-Mubarak H., Chen H.H., Adam M., Jain S., Narang T., Abdin R.M., Qadura M. (2018). Valproic Acid Induces Endothelial-to-Mesenchymal Transition-Like Phenotypic Switching. Front. Pharmacol..

[186] Chan T.S., Hsu C.C., Pai V.C., Liao W.Y., Huang S.S., Tan K.T., Yen C.J., Hsu S.C., Chen W.Y., Shan Y.S. (2016). Metronomic chemotherapy prevents therapy-induced stromal activation and induction of tumor-initiating cells. J. Exp. Med..

[187] Su S., Chen J., Yao H., Liu J., Yu S., Lao L., Wang M., Luo M., Xing Y., Chen F. (2018). CD10^+^GPR77^+^ Cancer-Associated Fibroblasts Promote Cancer Formation and Chemoresistance by Sustaining Cancer Stemness. Cell.

[188] Kim J., Kim S., Park S.Y., Lee G.K., Lim K.Y., Kim J.Y., Hwang J.A., Yu N., Kang E.H., Hwang M. (2023). Molecular Subtypes and Tumor Microenvironment Characteristics of Small-Cell Lung Cancer Associated with Platinum-Resistance. Cancers.

[189] Sohal S.S. (2017). Epithelial and endothelial cell plasticity in chronic obstructive pulmonary disease (COPD). Respir. Investig..

[190] Cheng Q., Zhou L., Zhou J., Wan H., Li Q., Feng Y. (2016). ACE2 overexpression inhibits acquired platinum resistance-induced tumor angiogenesis in NSCLC. Oncol. Rep..

[191] Song Y., Lee S.Y., Kim A.R., Kim S., Heo J., Shum D., Kim S.H., Choi I., Lee Y.J., Seo H.R. (2018). Identification of radiation-induced EndMT inhibitors through cell-based phenomic screening. FEBS Open Bio..

[192] Choi K.J., Nam J.K., Kim J.H., Choi S.H., Lee Y.J. (2020). Endothelial-to-mesenchymal transition in anticancer therapy and normal tissue damage. Exp. Mol. Med..

[193] Han R., Guo H., Shi J., Zhao S., Jia Y., Liu X., Liu Y., Cheng L., Zhao C., Li X. (2024). Osimertinib in combination with antiangiogenesis therapy presents a promising option for osimertinib-resistant non-small cell lung cancer. BMC Med..

[194] Abbona A., Paccagnella M., Astigiano S., Martini S., Denaro N., Ruatta F., Barbieri O., Merlano M., Garrone O. (2022). Effect of Eribulin on Angiogenesis and the Expression of Endothelial Adhesion Molecules. Anticancer. Res..

[195] Masaki N., Wu N.F., Aoki Y., Yamamoto J., Miyazaki J., Hoffman R.M. (2021). Osteosarcoma of the Breast in a Patient Derived Orthotopic Xenograft (PDOX) Mouse Model Is Arrested by both Cisplatinum and Eribulin. In Vivo.

[196] Takahashi M., Kikawa Y., Kashiwabara K., Taira N., Iwatani T., Shimozuma K., Ohtani S., Yoshinami T., Watanabe J., Kashiwaba M. (2024). Eribulin versus S-1 as first or second-line chemotherapy to assess health-related quality of life and overall survival in HER2-negative metastatic breast cancer (RESQ study): A non-inferiority, randomised, controlled, open-label, phase 3 trial. EClinicalMedicine.

[197] Ito K., Hamamichi S., Abe T., Akagi T., Shirota H., Kawano S., Asano M., Asano O., Yokoi A., Matsui J. (2017). Antitumor effects of eribulin depend on modulation of the tumor microenvironment by vascular remodeling in mouse models. Cancer Sci..

[198] Funahashi Y., Okamoto K., Adachi Y., Semba T., Uesugi M., Ozawa Y., Tohyama O., Uehara T., Kimura T., Watanabe H. (2014). Eribulin mesylate reduces tumor microenvironment abnormality by vascular remodeling in preclinical human breast cancer models. Cancer Sci..

[199] Zhuo D., Mei Y., Lin C., Wu A., Luo Y., Lu H., Fu J. (2024). Nudifloside, a Secoiridoid Glucoside Derived from Callicarpa nudiflora, Inhibits Endothelial-to-Mesenchymal Transition and Angiogenesis in Endothelial Cells by Suppressing Ezrin Phosphorylation. J. Cancer.

[200] Calcabrini A., García-Martínez J.M., González L., Tendero M.J., Ortuño M.T., Crateri P., Lopez-Rivas A., Arancia G., González-Porqué P., Martín-Pérez J. (2006). Inhibition of proliferation and induction of apoptosis in human breast cancer cells by lauryl gallate. Carcinogenesis.

[201] Chu K.V., Fan C.S., Chen C.C., Hsieh S.C., Huang T.S. (2019). Octyl gallate induces pancreatic ductal adenocarcinoma cell apoptosis and suppresses endothelial-mesenchymal transition-promoted M2-macrophages, HSP90α secretion, and tumor growth. Cells.

[202] Wawro M.E., Sobierajska K., Ciszewski W.M., Niewiarowska J. (2019). Nonsteroidal Anti-Inflammatory Drugs Prevent Vincristine-Dependent Cancer-Associated Fibroblasts Formation. Int. J. Mol. Sci..

[203] Herbertz S., Sawyer J.S., Stauber A.J., Gueorguieva I., Driscoll K.E., Estrem S.T., Cleverly A.L., Desaiah D., Guba S.C., Benhadji K.A. (2015). Clinical development of galunisertib (LY2157299 monohydrate), a small molecule inhibitor of transforming growth factor-beta signaling pathway. Drug Des. Devel Ther..

[204] Yu J., Du X., Zhang S., Long J., Wu P., Li Z., Lyu X., Hong Q., Chen P., Gao B. (2024). Galunisertib promotes bevacizumab-induced vascular normalization in nasopharyngeal carcinoma: Multi-parameter MRI evaluation. Mol. Ther. Oncol..

[205] Liu S., Xu D.S., Li M., Zhang Y., Li Q., Li T.T., Ren L.Q. (2020). Icariin attenuates endothelial-mesenchymal transition via H19/miR-148b-3p/ELF5 in ox-LDL-stimulated HUVECs. Mol. Ther. Nucleic Acids..

[206] Li J., Wu X., Ji X.B., He C., Xu S., Xu X. (2023). Biphasic function of GSK3β in gefitinib-resistant NSCLC with or without EGFR mutations. Exp. Ther. Med..

[207] Kim K., Sohn Y.J., Lee R., Yoo H.J., Kang J.Y., Choi N., Na D., Yeon J.H. (2020). Cancer-Associated Fibroblasts Differentiated by Exosomes Isolated from Cancer Cells Promote Cancer Cell Invasion. Int. J. Mol. Sci..

[208] Kovacs R.J., Maldonado G., Azaro A., Fernández M.S., Romero F.L., Sepulveda-Sánchez J.M., Corretti M., Carducci M., Dolan M., Gueorguieva I. (2015). Safety of TGF-β Receptor I Kinase Inhibitor LY2157299 Monohydrate in Cancer Patients in a First-in-Human Dose Study. Cardiovasc. Toxicol..

[209] Liu D., van der Zalm A.P., Koster J., Bootsma S., Oyarce C., van Laarhoven H.W.M., Bijlsma M.F. (2024). Predictive biomarkers for response to TGF- β inhibition in resensitizing chemo(radiated) esophageal adenocarcinoma. Pharmacol. Res..

[210] Formenti S.C., Hawtin R.E., Dixit N., Evensen E., Lee P., Goldberg J.D., Li X., Vanpouille-Box C., Schaue D., McBride W.H. (2019). Baseline T cell dysfunction by single cell network profiling in metastatic breast cancer patients. J. Immunother. Cancer.

[211] Pantziarka P., André N. (2019). Editorial: Drug Repurposing. Front. Med..

[212] Pantziarka P., Verbaanderd C., Huys I., Bouche G., Meheus L. (2021). Repurposing drugs in oncology: From candidate selection to clinical adoption. Semin. Cancer Biol..

[213] Verbaanderd C., Meheus L., Huys I., Pantziarka P. (2017). Repurposing Drugs in Oncology: Next Steps. Trends Cancer.

[214] Wylie-Sears J., Levine R.A., Bischoff J. (2014). Losartan inhibits endothelial-to-mesenchymal transformation in mitral valve endothelial cells by blocking transforming growth factor-β-induced phosphorylation of ERK. Biochem. Biophys. Res. Commun..

[215] Yao J., Guihard P.J., Blazquez-Medela A.M., Guo Y., Moon J.H., Jumabay M., Boström K.I., Yao Y. (2015). Serine Protease Activation Essential for Endothelial-Mesenchymal Transition in Vascular Calcification. Circ. Res..

[216] Guo Y., Li P., Bledsoe G., Yang Z.R., Chao L., Chao J. (2015). Kallistatin inhibits TGF-beta-induced endothelial-mesenchymal transition by differential regulation of microRNA-21 and eNOS expression. Exp. Cell Res..

[217] Melisi D., Garcia-Carbonero R., Macarulla T., Pezet D., Deplanque G., Fuchs M., Trojan J., Kozloff M., Simionato F., Cleverly A. (2019). TGFβ receptor inhibitor galunisertib is linked to inflammation- and remodeling-related proteins in patients with pancreatic cancer. Cancer Chemother. Pharmacol..

[218] Faivre S., Santoro A., Kelley R.K., Gane E., Costentin C.E., Gueorguieva I., Smith C., Cleverly A., Lahn M.M., Raymond E. (2019). Novel transforming growth factor beta receptor I kinase inhibitor galunisertib (LY2157299) in advanced hepatocellular carcinoma. Liver Int..

[219] Formenti S.C., Lee P., Adams S., Goldberg J.D., Li X., Xie M.W., Ratikan J.A., Felix C., Hwang L., Faull K.F. (2018). Focal Irradiation and Systemic TGFβ Blockade in Metastatic Breast Cancer. Clin. Cancer Res..

[220] Morris J.C., Tan A.R., Olencki T.E., Shapiro G.I., Dezube B.J., Reiss M., Hsu F.J., Berzofsky J.A., Lawrence D.P. (2014). Phase I study of GC1008 (fresolimumab): A human anti-transforming growth factor-beta (TGFβ) monoclonal antibody in patients with advanced malignant melanoma or renal cell carcinoma. PLoS ONE.

[221] Rice L.M., Padilla C.M., McLaughlin S.R., Mathes A., Ziemek J., Goummih S., Nakerakanti S., York M., Farina G., Whitfield M.L. (2015). Fresolimumab treatment decreases biomarkers and improves clinical symptoms in systemic sclerosis patients. J. Clin. Investig..

[222] Vincenti F., Fervenza F.C., Campbell K.N., Diaz M., Gesualdo L., Nelson P., Praga M., Radhakrishnan J., Sellin L., Singh A. (2017). Focal Segmental Glomerulosclerosis Study Group. A Phase 2, Double-Blind, Placebo-Controlled, Randomized Study of Fresolimumab in Patients With Steroid-Resistant Primary Focal Segmental Glomerulosclerosis. Kidney Int. Rep..

[223] Shenderov K., Collins S.L., Powell J.D. (2021). Horton MR. Immune dysregulation as a driver of idiopathic pulmonary fibrosis. J. Clin. Investig..

[224] Mascarenhas J., Li T., Sandy L., Newsom C., Petersen B., Godbold J., Hoffman R. (2014). Anti-transforming growth factor-β therapy in patients with myelofibrosis. Leuk. Lymphoma.

